# Critical care access and outcomes in residents of inpatient-care catchment areas with and without ICUs: a nationwide cohort study in Japan

**DOI:** 10.1016/j.lanwpc.2026.101868

**Published:** 2026-04-30

**Authors:** Hiroyuki Ohbe, Nobuaki Shime, Kei Nishiyama, Takuya Sato, Kent Doi, Kiyohide Fushimi, Hiroki Matsui, Hideo Yasunaga, Daisuke Kudo

**Affiliations:** a Department of Emergency and Critical Care Medicine, Tohoku University Hospital, 1-1 Seiryo-machi, Aoba-ku, Sendai 980-8574, Japan; b Department of Clinical Epidemiology and Health Economics, School of Public Health, The University of Tokyo, 7-3-1 Hongo, Bunkyo-ku, Tokyo 113-0033, Japan; c Department of Emergency and Critical Care Medicine, Graduate School of Biomedical & Health Sciences, Hiroshima University, 1-2-3 Kasumi, Minami-Ku, Hiroshima 734-8551, Japan; d Department of Emergency and Critical Care, Niigata University, 1-754 Asahimachidori, Chuo-ku, Niigata 951-8510, Japan; e Department of Emergency and Critical Care Medicine, The University of Tokyo Hospital, 7-3-1 Hongo, Bunkyo-ku, Tokyo 1138655, Japan; f Department of Health Policy and Informatics, Institute of Science Tokyo Graduate School, 2-12-1 Ookayama, Meguro-ku, Tokyo 152-8550, Japan; g Division of Emergency and Critical Care Medicine, Tohoku University Graduate School of Medicine, 2-1 Seiryo-machi, Aoba-ku, Sendai, Miyagi 980-8575, Japan

**Keywords:** Intensive care units, Health services accessibility, Healthcare disparities, Secondary medical areas, Japan

## Abstract

**Background:**

In Japan, many secondary medical areas (SMAs), defined as regional self-sufficient inpatient-care planning units, are not equipped with intensive care unit (ICU) beds. We aimed to describe the national distribution of SMAs without ICUs, compared post-arrival outcomes among ICU-admitted patients, and evaluated ICU access and mortality among critically ill patients.

**Methods:**

This retrospective cohort study linked data from the Diagnosis Procedure Combination database with the Hospital Bed Function Report of 2022. The primary exposure was residence in an SMA without ICU beds. Outcomes were assessed using multivariate generalized linear models.

**Findings:**

Among 335 SMAs, 140 (41.8%) lacked ICU beds, encompassing 11.6% of the national population and 46.0% of the land area. Among the 282,894 ICU-admitted patients, residents of SMAs without ICUs travelled substantially farther to reach an ICU (median 32.4 vs. 5.7 km), but had no statistically significant difference in adjusted in-hospital mortality (adjusted risk difference, −0.38 percentage points; 95% confidence interval [CI], −0.81 to 0.05). In a separate analysis of 467,200 critically ill patients, those living in SMAs without ICUs had lower ICU admission rates (24.9% vs. 35.6%) and statistically significant but small increase in adjusted in-hospital mortality (adjusted risk difference +0.96 percentage points; 95% CI: 0.17–1.75).

**Interpretation:**

Nearly half of SMAs lack ICU beds. While post-arrival ICU outcomes were similar, residence in an SMA without ICUs was associated with reduced ICU access and higher mortality among critically ill patients.

**Funding:**

Ministry of Health, Labor and Welfare, Japan; and Japan Agency for Medical Research and Development.

**Research in context**

**Evidence before this study**

We searched PubMed for studies published from database inception to April 9, 2026, without language restrictions, using combinations of Medical Subject Headings (MeSH) related to intensive care (Intensive Care Units, Critical Care), health services accessibility and referral pathways (Health Services Accessibility, Patient Transfer, Referral and Consultation), and health-care resources and regional planning (Health Resources, Regional Health Planning). Previous work has documented geographic variation in ICU capacity and has examined associations between critical care resources, interhospital transfer, and outcomes. However, we found that population-based evidence explicitly characterising health-care catchment areas with no ICU beds and separating barriers to ICU access before admission from outcomes after ICU admission was limited.

**Added value of this study**

This nationwide cohort study evaluated disparities using secondary medical areas (SMAs) in Japan. Overall, 140 of 335 SMAs (41.8%) had no ICU beds, covering 11.6% of the population and 46.0% of the land area. Among 282,894 ICU-admitted patients, residents of SMAs without ICUs travelled substantially farther to reach an ICU, yet had no statistically significant difference in adjusted in-hospital mortality compared with residents of SMAs with ICUs. In contrast, among 467,200 critically ill adults with guideline-based indications for ICU admission, residence in an SMA without ICUs was associated with lower ICU admission and a small but statistically significant increase in adjusted in-hospital mortality.

**Implications of all the available evidence**

Our findings suggest that the disadvantage associated with living in an area without ICU beds is driven primarily by limited access to ICU admission, rather than quality of care once ICU care is reached. These findings suggest that improving pre-arrival access in low-density SMAs without any ICU beds and selectively strengthening ICU capacity in high-density SMAs with relatively low ICU bed availability may offer more feasible strategies than uniformly establishing new ICUs across all SMAs.

## Introduction

Intensive care units (ICUs) form a fundamental component of modern healthcare systems, providing organ support and advanced monitoring for critically ill patients.^1^ Therefore, access to ICU care is an important consideration in regional healthcare planning; however, substantial geographical disparities in ICU availability have been reported worldwide,^2^, ^3^, ^4^, ^5^ and also in Japan.^6^, ^7^, ^8^ The secondary medical area (SMA), a regional planning unit established under the universal health insurance coverage system, is responsible for organizing comprehensive inpatient care.^9^ SMAs are intended to function as self-sufficient, basic geographic units through which residents can access essential inpatient care services. However, some SMAs lack ICU bed infrastructure, suggesting that the residents of these areas may face fundamental barriers to critical care access. However, to the best of our knowledge, no prior study has directly evaluated regions in which an entire legally defined inpatient-care catchment areas lacks ICU capacity, either in Japan or internationally.

Therefore, we conducted a nationwide cohort study whose primary aims were to: (1) describe the national distribution and population burden of SMAs with no ICU beds; (2) examine whether post-arrival clinical outcomes differed for ICU-admitted patients by residential ICU availability; and (3) evaluate how living in an SMA without ICUs affects ICU access, travel distance, and mortality among critically ill adults with guideline-based indications for intensive care. The findings of this work will guide policymakers devise regional strategies to ensure more equitable access to critical care.

## Methods

### Data source

This nationwide retrospective cohort study used two data sources: the Diagnosis Procedure Combination (DPC) Study Group database from April 1, 2022, to March 31, 2023, and the Hospital Bed Function Report for fiscal year 2022 in Japan.^10^^,^^11^ The DPC Study Group database includes discharge summaries and administrative claims from more than 1500 voluntarily participating acute-care hospitals, representing approximately 50% of all acute-care beds nationwide.^10^ The database consolidates detailed patient-level data for all hospitalizations, including demographics, diagnoses, daily medical reimbursement codes, and discharge status. A previous study revealed that the sensitivity and specificity of interventions recorded in the DPC exceeded 90% each, whereas the sensitivity and specificity of the primary diagnoses were 78.9% and 93.2%, respectively.^12^

The Hospital Bed Function Report is an annual, mandatory hospital-reported survey published by the Ministry of Health, Labor, and Welfare of Japan, and provides facility-level information on hospital functions, bed categories, and facility characteristics as of July 1 each year.^11^ The report contains facility-level information only and does not include any patient-level data. Both the DPC database and the Hospital Bed Function Report are reported by hospitals according to standardized national data specifications.^10^^,^^11^ We linked the two datasets at the hospital (center) level using the hospital identifier/code recorded in both sources.

### Geographical unit and exposure

The geographical unit of analysis was the SMA, the principal administrative unit for organizing regional inpatient healthcare delivery under the Medical Care Act of Japan, and a geographically defined catchment area (not a hospital).^13^^,^^14^ Japan has 335 SMAs, which serve as the primary planning units for ensuring access to essential inpatient services, including emergency and critical care services.^13^^,^^14^ In contrast, 52 tertiary medical areas—typically corresponding to prefectural boundaries—are designated to provide highly specialized medical care such as organ transplantation, and treatment for extensive burns or rare diseases.^13^ Accordingly, standard critical care interventions (e.g., invasive mechanical ventilation [IMV], vasopressor support, and mechanical circulatory support [MCS]) are generally expected to be available within an SMA through local provision or established referral/transfer pathways.

Patients are assigned to an SMA based on the postal code of their residence. We defined the primary exposure as residence in an SMA without ICUs (zero ICU-designated beds) or an SMA with ICUs (at least one ICU-designated bed). The exposure assignment and subsequent care pathways captured and categorized in this study are summarized schematically in Supplementary Fig. S1.

### Definitions of the ICU and IMCU

Under the universal health insurance coverage system in Japan, ICUs are defined as specialized units providing critical care services with intensivist requirements and a nurse-to-patient ratio of ≤1:2.^15^ Further details on the definitions and relevant reimbursement codes of ICU and intermediate care units (IMCU) are provided in the Supplementary Methods and Supplementary Table S1.

### Study design, analytic cohorts, and outcomes

We employed three prespecified analyses to comprehensively evaluate the regional disparities in critical care.

First, we quantified the distribution of SMAs without ICUs using the Hospital Bed Function Report.^11^ For each SMA, we calculated the population (2020 Population Census),^16^ land area (National Land Numerical Information),^17^ and bed supply (Hospital Bed Function Report 2022),^11^ and visualized geographic patterns using choropleth maps. We also visualized the relationship between SMA population size and ICU bed density using a scatter plot.

Second, to evaluate whether the outcomes after reaching the ICU differed between residents of SMAs with vs. without ICUs, we identified all patients admitted to an ICU from the DPC Study Group database. We excluded patients admitted to hospitals that could not be linked to the Hospital Bed Function Report 2022 and patients whose residential addresses were unavailable. The primary outcome was in-hospital mortality. The secondary outcomes included ICU mortality, length of ICU stay, length of hospital stay, and total hospitalization costs.

Third, to assess the structural barriers to ICU access among patients for whom ICU admission is explicitly recommended, we identified critically ill patients who received organ support or advanced monitoring irrespective of ICU admission from the DPC Study Group database, as defined by the ICU admission and discharge guideline devised by the Japanese Society of Intensive Care Medicine.^15^ This guideline is conceptually aligned with global ICU-triage principles.^2^ The inclusion criteria were the use of any of the following: (1) IMV, (2) MCS (intra-aortic balloon pumping, extracorporeal membrane oxygenation, Impella, or ventricular assist device), (3) vasopressors (dopamine, dobutamine, noradrenaline, adrenaline, or vasopressin), (4) continuous renal replacement therapy (CRRT), or (5) advanced monitoring (pulmonary artery catheter monitoring, cardiac output monitoring, targeted temperature management, or intracranial pressure monitoring). We excluded patients admitted to hospitals that could not be linked to the Hospital Bed Function Report 2022 and patients whose residential addresses were unavailable. The co-primary outcomes were ICU admission during hospitalization and in-hospital mortality. The secondary outcomes were intermediate care unit (IMCU) admission, no ICU/IMCU admission during hospitalization, length of hospital stay, total hospitalization costs, and early hospital transfer within 2 days of initiation of organ support or advanced monitoring.

Travel distance was defined as the straight-line distance from the patient’s residential postal-code area to the treating hospital: the ICU-admitting hospital in the ICU-admitted cohort, and the hospital where organ support/advanced monitoring was initiated in the critically ill cohort.

### Covariates

We adjusted for the following patient demographics in all the multivariate models: age, sex, Charlson Comorbidity Index, level of independence before hospitalization, location before hospitalization, admission classification, level of consciousness on admission (Japan Coma Scale), main etiologies for admission, receipt of cardiopulmonary resuscitation, and receipt of organ support therapy. Baseline characteristics were assessed at hospital admission. In contrast, cardiopulmonary resuscitation and organ support therapy were defined on the day of ICU admission (ICU-admitted cohort) or on the day of initiation of organ support or advanced monitoring (critically ill cohort). We included procedure-based organ support therapies as severity proxies to capture organ failure in administrative data, in line with a previously developed and validated procedure-based organ failure assessment model for ICU patients in the DPC database.^18^

### Statistical analysis

Continuous variables were summarized as medians with interquartile ranges, and categorical variables as numbers and percentages. Standardized mean differences (SMD) were used to compare characteristics, with SMD values < 0.1 denoting negligible imbalance between the groups.

To evaluate the association between residence in a SMA without ICUs and each outcome, we generated generalized linear models with a Gaussian distribution and an identity link function, irrespective of whether the outcome was binary or continuous. We reported both unadjusted and adjusted risk differences. For unadjusted comparisons, we fit models including only the exposure variable (residential SMA type). For adjusted comparisons, we included all covariates described above. Risk differences were defined as (SMAs without ICUs − SMAs with ICUs), with SMAs with ICUs as the reference category. Cluster-robust standard errors were applied to account for patient-level clustering within hospitals.

We conducted four sensitivity analyses. First, we assessed dose–response relationships by categorizing residential SMAs into five groups according to ICU beds per 100,000 population (0, 0.1–2.9, 3.0–5.9, 6.0–8.9, and 9.0–27.9). Second, to examine whether the associations differed by SMA population size, we performed a population-stratified analysis. Because all SMAs with a population of ≥400,000 had at least one ICU bed (i.e., no exposure overlap), this analysis was restricted to SMAs with a population <400,000 and compared SMAS with a population of <200,000 vs. 200,000–399,999. Third, in the critically ill cohort, we performed analyses excluding receipt of organ support therapy from the adjustment set to evaluate potential attenuation due to conditioning on downstream consequences of delayed access. Fourth, in the critically ill cohort, we performed analyses excluding patients who met the critically ill definition solely by dopamine infusion, dobutamine infusion, or advanced monitoring (without any other qualifying organ support).

Subgroup analyses were used to examine effect heterogeneity across organ-support modalities (IMV, vasopressor, MCS, CRRT and advanced monitoring) and by admission classification (elective surgery vs. emergency surgery/non-surgery). We focused on the primary outcomes for the sensitivity and subgroup analyses.

Two-sided P-values < 0.05 were considered statistically significant. All analyses were conducted using Stata/SE version 19.0 (StataCorp, College Station, TX, USA).

### Ethical approval

This study was approved by the Institutional Review Board of the University of Tokyo (approval number: 3501-5; approval date: May 19, 2021). As all data were de-identified, the requirement for informed consent was waived. This study adhered to the tenets of the Declaration of Helsinki.

### Role of the funding source

The funders had no role in study design, data collection, data analysis, data interpretation, writing of the report, or the decision to submit for publication. HO had full access to all the data and had final responsibility for the decision to submit for publication.

## Results

**Fig. 1:**
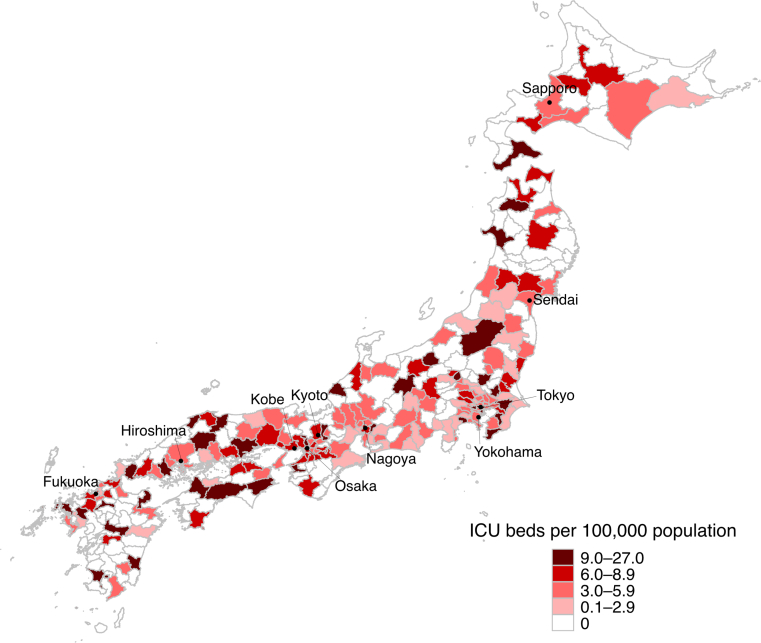
Geographic distribution of ICU beds per 100,000 population across secondary medical areas in Japan. Secondary medical areas without ICU beds are shown in white. The color intensity reflects ICU bed density per 100,000 populations. ICU = intensive care unit.

A total of 335 SMAs were included in the geographical analysis. Of these, 140 SMAs (41.8%) lacked ICUs (Table 1). Nationally, 11.6% of the population resided in SMAs without ICUs. These regions accounted for 46.0% of Japan’s land area, with a markedly low population density (median 86,000 vs. 506,000 persons/km^2^ for SMAs without and with ICUs, respectively). All SMAs without ICUs had populations <400,000 (Supplementary Fig. S2). SMAs without ICUs had slightly fewer acute-care beds per population but a higher density of total hospital beds. Geographic visualization demonstrated pronounced spatial inequities; SMAs without ICUs were clustered in sparsely populated regions such as Hokkaido, Tohoku, Hokuriku, Shikoku, and Kyushu (Fig. 1). Geographic distribution of ICU bed density by reimbursement-based ICU classification are shown in Supplementary Figs. S3–S5. Characteristics of SMAs stratified by ICU bed density are summarized in Supplementary Table S2.

**Table 1:** Characteristics of secondary medical areas (SMAs) stratified by ICU availability.

	Overall	SMAs	SMAs	SMD
		without ICUs	with ICUs	%
Number of SMA	335	140 (41.8%)	195 (58.2%)	
Population, thousand persons				
Median (IQR)	216 (96–466)	83 (55–137)	428 (242–722)	1.28
Total	125,555	14,534 (11.6%)	111,021 (88.4%)	–
Population category, n (%)				
19,219–199,999	161 (48.1%)	125 (89.3%)	36 (18.5%)	−2.01
200,000–399,999	69 (20.6%)	15 (10.7%)	54 (27.7%)	0.44
400,000–599,999	41 (12.2%)	0 (0.0)	41 (21.0%)	0.73
600,000–3,758,300	64 (19.1%)	0 (0.0)	64 (32.8%)	0.99
Land area, km^2^				
Median (IQR)	855 (437–1413)	984 (569–1414)	731 (325–1413)	−0.18
Total	374,673	172,378 (46.0%)	202,295 (54.0%)	–
Population density, persons/km^2^				
Median (IQR)	233 (86–673)	86 (50–182)	506 (230–1794)	0.73
Number of ICU beds				
Median, IQR	6 (0–24)	0 (0–0)	20 (10–46)	1.19
Total	6933	0 (0.0%)	6933 (100.0%)	–
Per 100,000 population	2.5 (0–5.8)	0 (0–0)	5.3 (3.2–7.6)	2.16
Annual number of ICU patients				
Median, IQR	405 (0–1735)	0 (0–0)	1392 (567–2891)	1.20
Region with IMCU beds	239 (71.3%)	57 (40.7%)	182 (93.3%)	1.35
Number of IMCU beds				
Median, IQR	20 (0–57)	0 (0–9)	48 (25–94)	1.25
Total	14,399	863 (6.0%)	13,536 (94.0%)	–
Per 100,000 population	9.0 (0.0–14.2)	0.0 (0.0–9.8)	11.6 (7.5–15.7)	0.95
Number of acute-care hospital beds				
Median, IQR	1105 (468–2548)	399 (246–664)	2077 (1264–3455)	1.28
Total	621,922	68,571 (11.0%)	553,351 (89.0%)	–
Per 100,000 population	496 (392–615)	470 (370–582)	509 (403–629)	0.23
Number of all hospital beds				
Median, IQR	1949 (916–4115)	848 (558–1290)	3574 (2310–5837)	2.38
Total	1,065,972	137,293 (12.9%)	928,679 (87.1%)	–
Per 100,000 population	910 (719–1123)	954 (748–1176)	862 (708–1063)	−0.30
Academic hospital	66 (19.7%)	0 (0.0%)	66 (33.8%)	–
Tertiary emergency hospital	222 (66.3%)	46 (32.9%)	176 (90.3%)	1.46

For the “Total” rows, the values are the sums across SMAs; the percentages in parentheses indicate the share of the overall total (e.g., national population, land area, or total beds) accounted for by each SMA group.

SMD indicates the magnitude of between-group imbalance; values > 0.10 are commonly considered meaningful.

SMA = secondary medical area; ICU = intensive care unit; SMD = standardized mean difference; IMCU = intermediate care unit; IQR = interquartile range.

In fiscal year 2022, the DPC Study Group database covered 304,515/621,922 acute-care beds nationally (49.0%). Coverage differed by residential SMA type (34.3% in SMAs without ICU beds vs. 50.8% in SMAs with ICU beds).

Subsequently, among 316,036 ICU admissions captured in the DPC database, 282,894 (89.5%) were included in the ICU-admitted cohort (Table 2 and Supplementary Fig. S6), of whom, 21,434 patients (7.6%) resided in SMAs without ICUs. More than half of patients from SMAs without ICUs were admitted to ICUs located in SMAs with high ICU bed capacity (9.0–27.9 beds per 100,000 population; 53.6% [11,495/21,434]; Table 2). Travel distance from the residence to the admitting hospital was substantially greater for residents of SMAs without ICUs (median 32.4 km vs. 5.7 km). The baseline characteristics were generally similar between groups, although interhospital transfer and postoperative admissions were more common among patients from SMAs without ICUs. Unadjusted in-hospital mortality was lower in SMAs without ICUs than in SMAs with ICUs (8.7% vs. 13.1%; −4.42 percentage-points; 95% CI, −5.58 to −3.27; Table 3). After adjustment, the difference was small and not statistically significant (−0.38 percentage points; 95% CI, −0.81 to 0.05; Table 3); the full model specification is provided in Supplementary Table S3. Similarly, no significant adjusted differences were observed in ICU mortality, length of ICU and hospital stay, or total ICU and hospitalization costs (Table 3). Geographic distribution of SMA-level median travel distance and mean unadjusted in-hospital mortality in the ICU-admitted cohort are shown in Supplementary Figs. S7 and S8.

**Table 2:** Baseline characteristics of patients admitted to ICUs stratified by residential SMA type.

Variables	Overall, N = 282,894	Patients residing in SMAs without ICUs, n = 21,434	Patients residing in SMAs with ICUs, n = 261,460	SMD%
Geographical healthcare factors				
ICU beds per 100,000 population in residential SMA				
0	21,434 (7.6)	21,434 (100.0)	0 (0.0)	–
0.1–2.9	33,439 (11.8)	0 (0.0)	33,439 (12.8)	–
3.0–5.9	99,355 (35.1)	0 (0.0)	99,355 (38.0)	–
6.0–8.9	72,796 (25.7)	0 (0.0)	72,796 (27.8)	–
9.0–27.9	55,870 (19.7)	0 (0.0)	55,870 (21.4)	–
ICU beds per 100,000 population in hospital SMA				
0	0 (0.0)	0 (0.0)	0 (0.0)	–
0.1–2.9	15,516 (5.5)	619 (2.9)	14,897 (5.7)	−0.14
3.0–5.9	90,163 (31.9)	4513 (21.1)	85,650 (32.8)	−0.27
6.0–8.9	82,772 (29.3)	4807 (22.4)	77,965 (29.8)	−0.17
9.0–27.9	94,443 (33.4)	11,495 (53.6)	82,948 (31.7)	0.45
Hospital within the same SMA as residence	186,617 (66.0)	0 (0.0)	186,617 (71.4)	–
Distance from residence to hospital, km, mean (SD)	16.1 (51.3)	49.5 (83.7)	13.4 (46.6)	0.53
Distance from residence to hospital, km, median (IQR)	6.3 (3.1–14.0)	32.4 (20.6–51.5)	5.7 (2.9–11.6)	0.53
Patient characteristics				
Age, years	67.6 (18.5)	66.9 (18.4)	67.7 (18.5)	−0.04
Male	170,390 (60.2)	13,197 (61.6)	157,193 (60.1)	0.03
Charlson comorbidity index	1.1 (1.5)	1.2 (1.5)	1.1 (1.5)	0.07
Level of independence				
Independent	246,171 (87.0)	19,215 (89.6)	226,956 (86.8)	0.09
Mild dependence	22,344 (7.9)	1374 (6.4)	20,970 (8.0)	−0.06
Severe dependence	14,379 (5.1)	845 (3.9)	13,534 (5.2)	−0.06
Location before hospitalization				
Home	255,905 (90.5)	18,374 (85.7)	237,531 (90.8)	−0.16
Another hospital	19,410 (6.9)	2713 (12.7)	16,697 (6.4)	0.21
Nursing home	7579 (2.7)	347 (1.6)	7232 (2.8)	−0.08
Admission classification				
Elective surgery	113,667 (40.2)	10,914 (50.9)	102,753 (39.3)	0.24
Emergency surgery	49,490 (17.5)	4026 (18.8)	45,464 (17.4)	0.04
Non-surgery	119,737 (42.3)	6494 (30.3)	113,243 (43.3)	−0.27
Japan Coma Scale score at admission				
Alert	204,199 (72.2)	17,135 (79.9)	187,064 (71.5)	0.20
Dizziness	35,074 (12.4)	2086 (9.7)	32,988 (12.6)	−0.09
Somnolence	12,310 (4.4)	777 (3.6)	11,533 (4.4)	−0.04
Coma	31,311 (11.1)	1436 (6.7)	29,875 (11.4)	−0.17
Main etiologies for admission				
Cancer	63,759 (22.5)	5448 (25.4)	58,311 (22.3)	0.07
Acute coronary syndrome	29,615 (10.5)	2250 (10.5)	27,365 (10.5)	0.00
Aortic dissection or aneurysm	26,222 (9.3)	2793 (13.0)	23,429 (9.0)	0.13
Stroke	24,261 (8.6)	1518 (7.1)	22,743 (8.7)	−0.06
Acute abdominal diseases	21,815 (7.7)	1414 (6.6)	20,401 (7.8)	−0.05
Acute heart failure	16,346 (5.8)	841 (3.9)	15,505 (5.9)	−0.09
Trauma	14,233 (5.0)	957 (4.5)	13,276 (5.1)	−0.03
Post cardiac arrest	13,322 (4.7)	307 (1.4)	13,015 (5.0)	−0.20
Sepsis	12,367 (4.4)	831 (3.9)	11,536 (4.4)	−0.03
Pneumonia	3720 (1.3)	156 (0.7)	3564 (1.4)	−0.06
Aspiration	3722 (1.3)	166 (0.8)	3556 (1.4)	−0.06
CPR on the day of ICU admission	14,064 (5.0)	366 (1.7)	13,698 (5.2)	−0.19
Organ support therapy on the day of ICU admission				
Invasive mechanical ventilation	51,414 (18.2)	3426 (16.0)	47,988 (18.4)	−0.06
Intra-aortic balloon pumping	4393 (1.6)	370 (1.7)	4023 (1.5)	0.01
Extracorporeal membrane oxygenation	2530 (0.9)	175 (0.8)	2355 (0.9)	−0.01
Impella	962 (0.3)	84 (0.4)	878 (0.3)	0.01
Ventricular assist device	116 (0.0)	18 (0.1)	98 (0.0)	0.02
Dopamine	24,912 (8.8)	3243 (15.1)	21,669 (8.3)	0.21
Dobutamine	29,979 (10.6)	3671 (17.1)	26,308 (10.1)	0.21
Noradrenaline	87,761 (31.0)	8411 (39.2)	79,350 (30.3)	0.19
Adrenaline	14,460 (5.1)	1516 (7.1)	12,944 (5.0)	0.09
Vasopressin	7721 (2.7)	642 (3.0)	7079 (2.7)	0.02
Continuous renal replacement therapy	6185 (2.2)	533 (2.5)	5652 (2.2)	0.02
Pulmonary artery catheter monitoring	17,774 (6.3)	2101 (9.8)	15,673 (6.0)	0.14
Cardiac output monitoring	24,556 (8.7)	3214 (15.0)	21,342 (8.2)	0.21
Targeted temperature management	1960 (0.7)	103 (0.5)	1857 (0.7)	−0.03
Intracranial pressure monitoring	408 (0.1)	28 (0.1)	380 (0.1)	0.00

Baseline patient characteristics were assessed at hospital admission. Cardiopulmonary resuscitation and organ support therapy were assessed on the day of ICU admission.

SMD indicates the magnitude of between-group imbalance; values > 0.10 are commonly considered meaningful.

SMA = secondary medical area; ICU = intensive care unit; SMD = standardized mean difference; SD = standard deviation; IQR = interquartile range; CPR = cardiopulmonary resuscitation.

**Table 3:** Outcomes of patients admitted to ICUs stratified by residential SMA type.

Outcome	Values	Unadjusted risk difference (95% CI)	P value	Adjusted risk difference (95% CI)	P value
In-hospital mortality, %					
SMAs without ICUs	1868 (8.7)	−4.42 (−5.58 to −3.27)	<0.001	−0.38 (−0.81 to 0.05)	0.080
SMAs with ICUs	34,349 (13.1)	Ref.	–	Ref.	–
ICU mortality, %					
SMAs without ICUs	949 (4.4)	−3.80 (−4.79 to −2.82)	<0.001	−0.17 (−0.48 to 0.13)	0.267
SMAs with ICUs	21,518 (8.2)	Ref.	–	Ref.	–
Length of ICU stay, days					
SMAs without ICUs	2.0 (1.0–4.0)	0.10 (−0.05 to 0.25)	0.182	−0.06 (−0.17 to 0.05)	0.323
SMAs with ICUs	2.0 (1.0–4.0)	Ref.	–	Ref.	–
Length of hospital stay, days					
SMAs without ICUs	19.0 (12.0–32.0)	2.50 (1.27 to 3.73)	<0.001	0.67 (−0.49 to 1.82)	0.259
SMAs with ICUs	17.0 (10.0–30.0)	Ref.	–	Ref.	–
Total ICU costs, million yen					
SMAs without ICUs	0.4 (0.1–1.1)	0.06 (−0.04 to 0.17)	0.239	−0.05 (−0.14 to 0.05)	0.354
SMAs with ICUs	0.4 (0.2–1.1)	Ref.	–	Ref.	–
Total hospitalization costs, million yen					
SMAs without ICUs	2.7 (1.6–4.7)	0.63 (0.46 to 0.79)	<0.001	0.09 (−0.02 to 0.20)	0.094
SMAs with ICUs	2.1 (1.3–3.8)	Ref.	–	Ref.	–

Values represent n (%) or the median (IQR), as appropriate.

The adjusted models were adjusted for age, sex, Charlson Comorbidity Index, level of independence, location before hospitalization, admission classification, level of consciousness, main etiologies for admission, cardiopulmonary resuscitation on the day of ICU admission, and organ support therapy on the day of ICU admission.

Risk differences were defined as (SMAs without ICUs − SMAs with ICUs), with SMAs with ICUs as the reference category. Positive values indicate higher risk in SMAs without ICUs, and negative values indicate lower risk.

Costs are shown in JPY; for reference, we used the 2022 average exchange rate (1 USD = 131.43 JPY).

SMA = secondary medical area; ICU = intensive care unit; CI = confidence interval.

Finally, among 517,450 hospitalizations meeting the screening definition for critical illness, 467,200 (90.3%) were included in the critically ill cohort (Supplementary Fig. S9). We found that 54,952 patients (11.8%) resided in SMAs without ICUs, 21,743 (39.6%) of whom received treatment at hospitals located within the same SMA, and the travel distances were much greater for these patients compared with those living in SMAs with ICUs (median 17.9 km vs. 5.3 km; Table 4). Although the demographic and clinical characteristics were broadly similar, patients from SMAs without ICUs had fewer post-cardiac arrest events and greater dopamine use than those from SMAs with ICUs. Early inter-hospital transfer within 2 days of initiation of organ support or advanced monitoring was rare (overall 0.5%; 0.6% in residents of SMAs without ICU beds vs. 0.5% in residents of SMAs with ICU beds). Overall, 160,609 patients (34.4%) were admitted to an ICU. We present patient characteristics stratified by ICU admission status (Supplementary Table S4). Patients admitted to the ICU more often underwent elective or emergency surgery (and less often non-surgical admissions) and received higher-intensity therapies and advanced monitoring on the initiation day (e.g., MCS, CRRT, and invasive hemodynamic monitoring).

**Table 4:** Baseline characteristics of critically ill patients requiring organ support or advanced monitoring stratified by residential SMA type.

Variables	Overall, N = 467,200	Patients residing in SMAs without ICUs, N = 54,952	Patients residing in SMAs with ICUs, N = 412,248	SMD %
Geographical healthcare factors				
ICU beds per 100,000 population in residential SMA				
0	54,952 (11.8)	54,952 (100.0)	0 (0.0)	–
0.1–2.9	63,614 (13.6)	0 (0.0)	63,614 (15.4)	–
3.0–5.9	164,300 (35.2)	0 (0.0)	164,300 (39.9)	–
6.0–8.9	113,255 (24.2)	0 (0.0)	113,255 (27.5)	–
9.0–27.9	71,079 (15.2)	0 (0.0)	71,079 (17.2)	–
ICU beds per 100,000 population in hospital SMA				
0	24,893 (5.3)	22,715 (41.3)	2178 (0.5)	1.16
0.1–2.9	45,331 (9.7)	1756 (3.2)	43,575 (10.6)	−0.29
3.0–5.9	157,250 (33.7)	7952 (14.5)	149,298 (36.2)	−0.52
6.0–8.9	129,359 (27.7)	7563 (13.8)	121,796 (29.5)	−0.39
9.0–27.9	110,367 (23.6)	14,966 (27.2)	95,401 (23.1)	0.09
Hospital within the same SMA as residence	339,469 (72.7)	21,743 (39.6)	317,726 (77.1)	−0.82
Distance from residence to hospital, km, mean (SD)	14.2 (46.3)	31.0 (62.2)	12.0 (43.3)	0.36
Distance from residence to hospital, km, median (IQR)	5.8 (2.9–12.4)	17.9 (7.2–35.5)	5.3 (2.7–10.5)	0.36
Patient characteristics				
Age, years	68.5 (21.6)	70.1 (20.1)	68.3 (21.8)	0.09
Male	274,685 (58.8)	32,627 (59.4)	242,058 (58.7)	0.01
Charlson Comorbidity Index	1.1 (1.5)	1.3 (1.6)	1.1 (1.5)	0.10
Level of independence				
Independent	380,112 (81.4)	43,807 (79.7)	336,305 (81.6)	−0.05
Mild dependence	48,096 (10.3)	6052 (11.0)	42,044 (10.2)	0.03
Severe dependence	38,992 (8.3)	5093 (9.3)	33,899 (8.2)	0.04
Location before hospitalization				
Home	410,332 (87.8)	46,656 (84.9)	363,676 (88.2)	−0.10
Another hospital	31,600 (6.8)	5128 (9.3)	26,472 (6.4)	0.11
Nursing home	25,268 (5.4)	3168 (5.8)	22,100 (5.4)	0.02
Admission classification				
Elective surgery	128,197 (27.4)	16,502 (30.0)	111,695 (27.1)	0.07
Emergency surgery	71,684 (15.3)	8427 (15.3)	63,257 (15.3)	0.00
Non-surgery	267,319 (57.2)	30,023 (54.6)	237,296 (57.6)	−0.06
Japan Coma Scale score at admission				
Alert	322,214 (69.0)	39,444 (71.8)	282,770 (68.6)	0.07
Dizziness	56,533 (12.1)	6710 (12.2)	49,823 (12.1)	0.00
Somnolence	19,185 (4.1)	2302 (4.2)	16,883 (4.1)	0.00
Coma	69,268 (14.8)	6496 (11.8)	62,772 (15.2)	−0.10
Main etiologies for admission				
Cancer	69,023 (14.8)	9043 (16.5)	59,980 (14.5)	0.05
Acute coronary syndrome	47,490 (10.2)	5862 (10.7)	41,628 (10.1)	0.02
Aortic dissection or aneurysm	26,481 (5.7)	3348 (6.1)	23,133 (5.6)	0.02
Stroke	23,083 (4.9)	2596 (4.7)	20,487 (5.0)	−0.01
Acute abdominal diseases	33,348 (7.1)	4254 (7.7)	29,094 (7.1)	0.03
Acute heart failure	43,215 (9.2)	5079 (9.2)	38,136 (9.3)	0.00
Trauma	20,624 (4.4)	2705 (4.9)	17,919 (4.3)	0.03
Post cardiac arrest	29,140 (6.2)	1848 (3.4)	27,292 (6.6)	−0.15
Sepsis	23,191 (5.0)	2601 (4.7)	20,590 (5.0)	−0.01
Pneumonia	9456 (2.0)	1256 (2.3)	8200 (2.0)	0.02
Aspiration	10,298 (2.2)	1173 (2.1)	9125 (2.2)	−0.01
CPR on the start day of organ support or advanced monitoring	45,164 (9.7)	4133 (7.5)	41,031 (10.0)	−0.09
Organ support therapy on the start day of organ support or advanced monitoring				
Invasive mechanical ventilation	162,621 (34.8)	17,201 (31.3)	145,420 (35.3)	−0.08
Intra-aortic balloon pumping	6327 (1.4)	797 (1.5)	5530 (1.3)	0.01
Extracorporeal membrane oxygenation	3163 (0.7)	238 (0.4)	2925 (0.7)	−0.04
Impella	1110 (0.2)	103 (0.2)	1007 (0.2)	−0.01
Ventricular assist device	577 (0.1)	89 (0.2)	488 (0.1)	0.01
Dopamine	79,741 (17.1)	12,552 (22.8)	67,189 (16.3)	0.17
Dobutamine	60,163 (12.9)	8318 (15.1)	51,845 (12.6)	0.07
Noradrenaline	240,040 (51.4)	27,441 (49.9)	212,599 (51.6)	−0.03
Adrenaline	65,574 (14.0)	6807 (12.4)	58,767 (14.3)	−0.05
Vasopressin	15,630 (3.3)	1657 (3.0)	13,973 (3.4)	−0.02
Continuous renal replacement therapy	8879 (1.9)	994 (1.8)	7885 (1.9)	−0.01
Pulmonary artery catheter monitoring	22,011 (4.7)	2657 (4.8)	19,354 (4.7)	0.01
Cardiac output monitoring	35,591 (7.6)	4926 (9.0)	30,665 (7.4)	0.06
Targeted temperature management	2748 (0.6)	190 (0.3)	2558 (0.6)	−0.04
Intracranial pressure monitoring	810 (0.2)	90 (0.2)	720 (0.2)	0.00

Baseline characteristics were assessed at hospital admission. Cardiopulmonary resuscitation and organ support therapy were assessed on the day of initiation of organ support or advanced monitoring.

SMD indicates the magnitude of between-group imbalance; values > 0.10 are commonly considered meaningful.

SMA = secondary medical area; ICU = intensive care unit; SMD = standardized mean difference; SD = standard deviation; IQR = interquartile range; CPR = cardiopulmonary resuscitation.

ICU admission occurred in 24.9% of residents of SMAs without ICUs compared with 35.6% of residents of SMAs with ICUs. After adjustment, living in an SMA without ICUs was associated with an 11.13 percentage-point lower probability of ICU admission (95% CI, −14.07 to −8.20; Table 5); the full model specification is provided in Supplementary Table S5. In-hospital mortality was 22.7% in SMAs without ICUs and 23.3% in SMAs with ICUs; after adjustment, residence in an SMA without ICUs was associated with a statistically significant but small increase in in-hospital mortality (+0.96 percentage points; 95% CI, 0.17 to 1.75; Table 5); the full model specification is provided in Supplementary Table S6. Residents of SMAs without ICUs also had higher rates of no ICU/IMCU admission, and longer hospital stays, whereas IMCU admission, total hospitalization costs, and early hospital transfer did not differ significantly between ICU-equipped and non-ICU SMAs. Geographic distribution of SMA-level median travel distance, mean ICU admission, and mean unadjusted in-hospital mortality in the critically ill cohorts are shown in Supplementary Figs. S10–S12.

**Table 5:** Outcomes of critically ill patients requiring organ support or advanced monitoring stratified by residential SMA type.

Outcome	Values	Unadjusted risk difference (95% CI)	P value	Adjusted risk difference (95% CI)	P value
ICU admission, %					
SMAs without ICUs	13,657 (24.9)	−10.79 (−14.59 to −7.00)	<0.001	−11.13 (−14.07 to −8.20)	<0.001
SMAs with ICUs	146,952 (35.6)	Ref.	–	Ref.	–
In-hospital mortality, %					
SMAs without ICUs	12,467 (22.7)	−0.57 (−2.74 to 1.61)	0.608	0.96 (0.17 to 1.75)	0.017
SMAs with ICUs	95,876 (23.3)	Ref.	–	Ref.	–
IMCU admission, %					
SMAs without ICUs	14,467 (26.3)	−4.20 (−7.51 to −0.89)	0.013	−2.89 (−5.97 to 0.19)	0.066
SMAs with ICUs	125,836 (30.5)	Ref.	–	Ref.	–
No ICU/IMCU admission, %					
SMAs without ICUs	29,234 (53.2)	12.81 (8.81 to 16.81)	<0.001	11.79 (8.48 to 15.09)	<0.001
SMAs with ICUs	166,489 (40.4)	Ref.		Ref.	
Length of hospital stay, days					
SMAs without ICUs	17.0 (8.0–33.0)	4.07 (1.69 to 6.45)	0.001	2.59 (0.35 to 4.84)	0.023
SMAs with ICUs	16.0 (7.0–31.0)	Ref.	–	Ref.	–
Total hospitalization costs, million yen					
SMAs without ICUs	1.9 (0.9–3.5)	0.056 (−0.127 to 0.240)	0.548	−0.049 (−0.166 to 0.069)	0.417
SMAs with ICUs	1.9 (0.9–3.5)	Ref.	–	Ref.	–
Early hospital transfer, %					
SMAs without ICUs	343 (0.62)	0.107 (−0.001 to 0.215)	0.052	0.111 (−0.010 to 0.233)	0.061
SMAs with ICUs	2131 (0.52)	Ref.	–	Ref.	–

Values represent n (%) or the median (IQR), as appropriate.

The adjusted models were adjusted for age, sex, Charlson Comorbidity Index, level of independence, location before hospitalization, admission classification, level of consciousness, main etiologies for admission, cardiopulmonary resuscitation on the day of ICU admission, and organ support therapy on the day of initiation of organ support or advanced monitoring.

Risk differences were defined as (SMAs without ICUs − SMAs with ICUs), with SMAs with ICUs as the reference category. Positive values indicate higher risk in SMAs without ICUs, and negative values indicate lower risk.

Costs are shown in JPY; for reference, we used the 2022 average exchange rate (1 USD = 131.43 JPY).

SMA = secondary medical area; ICU = intensive care unit; CI = confidence interval.

The ICU-admitted and critically ill cohorts partially overlapped, with 157,530 hospitalizations included in both cohorts (33.7% of the critically ill cohort and 55.7% of the ICU-admitted cohort).

The results of sensitivity and subgroup analyses are presented in Supplementary Tables S7 and S8. In ICU-admitted patients, the adjusted risk differences for in-hospital mortality were not statistically significant and were consistent across sensitivity and subgroup analyses (Supplementary Table S7).

In the critically ill cohort, higher residential ICU bed density was associated with higher ICU admission (24.9% vs. 42.8% across the lowest vs. highest categories). Statistically significant lower adjusted in-hospital mortality was observed only in categories with ≥6.0 ICU beds per 100,000 population (sensitivity analysis 1, Supplementary Table S8). In the population-stratified sensitivity analysis restricted to SMAs with <400,000 residents, statistically significant adjusted mortality differences were observed in the 200,000–399,999 population category but not in the 19,219–199,999 category (sensitivity analysis 2, Supplementary Table S8). In the critically ill cohort, excluding organ support at initiation from the adjustment set increased the adjusted mortality difference to +1.49 percentage points (sensitivity analysis 3, Supplementary Table S8). Overall, 15.9% of the critically ill cohort met the definition solely by dopamine (8.5%), dobutamine (4.7%), or advanced monitoring (2.6%). Excluding these patients did not materially change the adjusted results for ICU admission or in-hospital mortality (sensitivity analysis 4, Supplementary Table S8). In the subgroup analysis by admission classification, differences in mortality were not observed for elective surgical admissions, whereas the adjusted mortality was higher for emergency surgery/non-surgery (subgroup analysis 1, Supplementary Table S8). In the subgroup analysis by organ-support modality, larger reductions in ICU admission were observed among patients receiving IMV, MCS, or CRRT, and statistically significant differences in adjusted mortality were evident only in the IMV subgroup (subgroup analysis 2, Supplementary Table S8).

## Discussion

In this nationwide cohort study, three principal findings emerged. First, ICU beds in Japan are unevenly distributed, with 41.8% of SMAs having no ICU beds. Such SMAs accounted for 11.6% of the national population and 46.0% of the land area, signifying markedly low population density. Second, among patients who ultimately reached an ICU, residents of SMAs without ICUs travelled substantially farther to reach an ICU; however, in-hospital mortality and other clinical outcomes did not differ significantly between residents of SMAs with and without ICUs, suggesting substantial post-arrival equalization of the quality of ICU care. Third, among critically ill patients with guideline-based clinical indications for ICU admission, only 24.9% of residents of SMAs without ICUs received treatment in the ICU. Residents of SMAs without ICUs had substantially lower ICU admission rates and higher adjusted mortality, indicating substantial inequities in pre-arrival access to critical care that were accompanied by worse clinical outcomes.

Our study provides rare, internationally unique evidence by evaluating structural exposure, i.e., residents of SMAs without ICUs, establishing a robust framework for evaluating geographic inequities. To our knowledge, no prior studies have evaluated “residence in a region without an ICU” as the primary exposure. Furthermore, this study has several strengths, including the use of nationwide administrative data linked to a census of ICU capacity across all SMAs and the novel distinction between pre-arrival access and post-arrival outcomes.

Our geographical analysis demonstrated marked regional variation in ICU availability across Japan, with 140 (41.8%) SMAs lacking ICU bed infrastructure. Although multiple structural and historical factors likely contribute to this pattern, one plausible explanation is that ICU establishment in Japan is not mandated by law (e.g., the Medical Care Act) and has traditionally depended on individual hospital decisions. Consequently, ICUs tend to be concentrated in more populated regions, while large rural areas remain bereft of on-site intensive care capacity. Similar geographic disparities have been reported in the United States, where 37% of hospital service areas—mostly rural—had zero ICU beds, partly reflecting market forces in the absence of regulatory requirements for ICU provision.^19^ In contrast, several high-income countries incorporate ICU capacity into formal regional planning frameworks and referral networks (e.g., the United Kingdom, Australia, and the Netherlands).^20^, ^21^, ^22^

In our ICU-admission cohort, the adjusted in-hospital mortality and other clinical outcomes did not differ significantly between residents of SMAs with and without ICUs. This finding aligns with prior studies from the United States and United Kingdom, showing that once critically ill patients reach an ICU, mortality does not differ substantially across long vs. short travel distances or rural vs. urban regions.^23^, ^24^, ^25^ Several mechanisms may explain the absence of post-arrival outcome differences in Japan. First, critical care is standardized under the unified national reimbursement system, with protocolized management and consistent nursing ratios across hospitals. Second, 87.3% of residents of SMAs without ICUs were admitted directly to ICUs located in high-density SMAs, rather than being initially stabilized at small local hospitals, a pattern similar to that observed in the United States.^24^ Direct admission to higher-capacity ICUs likely reduces delays in initiating definitive critical care and avoids variation in early resuscitation quality across small hospitals. Notably, the lower crude (unadjusted) mortality among residents of SMAs without ICUs likely reflects case-mix and selection into the ICU-admitted cohort (i.e., patients must survive long enough to be transferred and admitted), as suggested by fewer post-cardiac arrest cases and a higher proportion of transfers in this group. Accordingly, these findings must be interpreted in the context of “arrival survivor bias,” as this analysis only included patients who successfully reached an ICU.^24^^,^^25^

Our study also showed that critically ill patients living in SMAs without ICUs had significantly lower ICU admission rates, higher general ward admission rates, longer travel distances, and higher adjusted mortality. These findings align with the mechanisms described in prior studies: compared with ward-based care, ICU admission improves survival for patients with a high severity of illness^26^^,^^27^; greater distance to definitive critical care contributes to treatment delays and higher mortality^28^; and limited ICU bed supply or capacity strain in the hospital increases ward management and worsens outcomes.^29^

Our findings indicate that the potential disadvantage associated with living in a region without an ICU occurs not from the differences in the quality of post-ICU arrival care, but rather from restrained access to ICU admission itself. Because the post-ICU arrival outcomes were comparable between residents of SMAs with and without ICUs, the absence of local ICU beds does not inherently imply inferior ICU care. Consequently, establishing new ICUs in every SMA—an approach that would be economically inefficient and operationally unsustainable in sparsely populated regions—may not be the most effective solution. Instead, our results point toward the necessity for system-level interventions that improve pre-arrival access, such as standardized ICU triage pathways, enhanced interfacility transfer systems, dedicated critical care transport teams, and the development of regionalized critical care networks.^1^^,^^20^^,^^22^ Importantly, our admission-type subgroup analyses suggest that the main disadvantage of living in an SMA without ICU beds is borne by emergency presentations, for whom outcomes are more likely to depend on timely triage, transport, and transfer to definitive critical care. In contrast, elective postoperative care can often be planned at ICU-equipped hospitals through established referral pathways, which may mitigate geographic disparities for elective patients.

At the same time, the frequency of ICU admission increased progressively with an increase in the ICU bed supply, and in-hospital mortality improved only in SMAs with ≥6.0 ICU beds per 100,000 population. Population-stratified analyses further suggested that mortality differences were observed in SMAs with populations of 200,000–399,999 but not in smaller SMAs (<200,000). Collectively, these findings support a dual-track policy approach: strengthening transfer-based systems for SMAs with populations <200,000 that lack ICU beds, while prioritizing ICU capacity expansion in SMAs with populations ≥200,000 to achieve an ICU bed density of ≥6.0 per 100,000 population (e.g., establishing ≥12 ICU beds in an SMA of 200,000 residents).

Some limitations of this study warrant consideration. First, because the DPC Study Group database is not a complete census and tends to overrepresent larger hospitals, smaller non-participating hospitals may be underrepresented, particularly in SMAs without ICU beds, where database coverage was lower. If these smaller hospitals have limited capacity to deliver ICU-level care or have fewer established pathways and resources for timely referral/transfer to ICU-equipped centers, our estimates may underestimate the true magnitude of ICU access disparities. Second, residual confounding is possible despite adjustment for extensive patient and hospital characteristics, although residential assignment is unlikely to be influenced by individual health status, reducing the risk of reverse association. Third, because patients who die before reaching the hospital or receiving organ support are not captured, the true magnitude of missed opportunities for critical care may exceed our estimates (“arrival survivor bias”). Fourth, delayed access to definitive critical care may worsen physiologic severity, thereby mediating the association between residential ICU unavailability and outcomes. As our primary models were adjusted for severity proxies, the total effect may have been attenuated; consistently, excluding organ support at initiation increased the adjusted mortality difference to +1.49 percentage points. Fifth, our definition of “critically ill patients with guideline-based indications” was defined using procedure-based initiation of organ support or advanced monitoring; because some therapies (e.g., vasopressors) may be delivered outside the ICU in monitored wards, this may overestimate ICU eligibility and bias estimates toward the null. However, the findings were unchanged in sensitivity analyses excluding patients identified solely by dopamine, dobutamine, or advanced monitoring, suggesting that this potential misclassification had limited impact on our main conclusions. Sixth, because we used straight-line distance, we could not account for travel time variability due to geography and transport conditions (e.g., mountainous regions, ferry transport, or weather), which may further exacerbate access inequities in some remote areas. Seventh, we could not ascertain do-not-resuscitate orders or other limitations of life-sustaining treatment in the administrative data. Eighth, because we could not longitudinally link patient trajectories across hospitals, we were unable to quantify baseline characteristics, time spent at the initial hospital prior to ICU admission, and post-discharge outcomes after the index hospitalization, and residual selection and unmeasured confounding may remain.

### Conclusion

In this nationwide cohort study, we identified substantial geographical inequities in the Japanese critical care system. Among patients who ultimately reached an ICU, residents of SMAs without ICUs travelled substantially farther to receive ICU care, however, adjusted in-hospital mortality did not differ significantly by residential ICU availability. In contrast, among critically ill patients with guideline-based indications for ICU admission, residents of SMAs without ICUs had lower ICU admission rates and a small but statistically significant increase in adjusted in-hospital mortality. These findings, together with our sensitivity and subgroup analyses, suggest that improving pre-arrival access in low-density SMAs without any ICU beds and selectively strengthening ICU capacity in high-density SMAs with relatively low ICU bed availability may offer more feasible strategies than uniformly establishing new ICUs across all SMAs. Our results may be relevant to other regionalized healthcare systems in which ICU establishment is not strictly mandated and ICU capacity is shaped largely by local decision-making.

## Contributors

HO conceived and designed the study. HO, KF, HM and HY processed the data. HO, TS, HM, and HY analyzed the data. HO and HY accessed and verified the underlying data. All authors interpreted the data. HO wrote the initial draft of the manuscript. All authors revised the manuscript for intellectual content and approved the final version. HO is the guarantor of this study. The corresponding author attests that all listed authors meet the authorship criteria and that no others meeting the criteria have been omitted.

## Data sharing statement

The data used in the manuscript cannot be made available because the datasets analyzed are not publicly available, owing to contracts with the hospitals that provided the data.

## Editor note

The Lancet Group takes a neutral position with respect to territorial claims in published maps and institutional affiliations.

## Declaration of interests

All authors declare no conflicts of interest to declare.
