# Supplementary material for: Critical care access and outcomes in residents of inpatient-care catchment areas with and without ICUs: a nationwide cohort study in Japan
Source: Lancet Reg Health West Pac. 2026 Apr 30;70:101868. doi: 10.1016/j.lanwpc.2026.101868 (PMC13146584; doi:10.1016/j.lanwpc.2026.101868)
Supplement: Supplementary Figs. S1–S12 and Tables S1–S8 [file mmc1.pdf]

Supplementary Materials

Critical Care Access and Outcomes in Residents of Inpatient-Care Catchment Areas With and Without ICUs: A Nationwide Cohort Study in Japan

List of Supplementary Materials

Supplementary Methods
Definitions of the ICU and IMCU

Supplementary Tables
Supplementary Table 1. Japanese Medical Reimbursement Codes Used To Define the ICU and IMCU in 2022
Supplementary Table 2. Characteristics of Secondary Medical Areas Stratified by ICU Availability Categories
Supplementary Table 3. Full Model Specification for In-hospital Mortality among Patients Admitted to ICUs
Supplementary Table 4. Characteristics of Critically Ill Patients Requiring Organ Support or Advanced Monitoring Stratified by ICU admission status
Supplementary Table 5. Full Model Specification for ICU Admission among Critically Ill Patients Requiring Organ Support or Advanced Monitoring
Supplementary Table 6. Full Model Specification for In-hospital Mortality among Critically Ill Patients Requiring Organ Support or Advanced Monitoring
Supplementary Table 7. Results of the Sensitivity and Subgroup Analyses in Patients Admitted to ICUs
Supplementary Table 8. Results of the Sensitivity and Subgroup Analyses in Critically Ill Patients Requiring Organ Support or Advanced Monitoring

Supplementary Figures
Supplementary Figure 1. Exposure Assignment and Subsequent Care Pathways Captured in This Study
Supplementary Figure 2. Relationship Between the Population Size of the Secondary Medical Area and ICU Bed Density
Supplementary Figure 3. Geographic Distribution of ICU Beds (A3011/A3012) per 100,000 Population Across Secondary Medical Areas in Japan
Supplementary Figure 4. Geographic Distribution of ICU Beds (A3013/A3014) per 100,000 Population Across Secondary Medical Areas in Japan
Supplementary Figure 5. Geographic Distribution of ICU Beds (A3002/A3004) per 100,000 Population Across Secondary Medical Areas in Japan
Supplementary Figure 6. Patient Flowchart for the ICU Admission Cohort
Supplementary Figure 7. Geographic Distribution of SMA-level median travel distance in the ICU-admitted cohort
Supplementary Figure 8. Geographic Distribution of SMA-level mean unadjusted in-hospital mortality in the ICU-admitted cohort
Supplementary Figure 9. Patient Flowchart for the Critically Ill Patient Cohort
Supplementary Figure 10. Geographic Distribution of SMA-level median travel distance in the critically ill cohorts
Supplementary Figure 11. Geographic Distribution of SMA-level mean unadjusted ICU admission in the critically ill cohorts
Supplementary Figure 12. Geographic Distribution of SMA-level mean unadjusted in-hospital mortality in the critically ill cohorts

Supplementary Methods

Definitions of the ICU and IMCU

Under the national health insurance in Japan, an ICU is defined as a separate unit providing critical care services with at least one on-site physician 24 h per day (at least two on-site intensivists 24 h per day only for reimbursement codes A3011 and A3012), around-the-clock nursing with a nurse-to-patient ratio of >1:2, an ICU nurse (A3011 and A3012 only; defined as a full-time, dedicated nurse with at least five years' experience in caring for critically ill patients, who has completed appropriate training, assigned to the ICU for at least 20 hours per week), a clinical engineer (A3011 and A3012 only; defined as a full-time, dedicated allied health professional responsible for the operation and maintenance of life-support equipment, and is present in the hospital 24 hours a day), and equipment necessary to care for critically ill patients.[1]

The definition of the IMCU is similar to that of the ICU, except that the IMCU does not require an intensivist, ICU nurse, or clinical engineer, and the requisite nurse-to-patient ratio is 1:3, 1:4, or 1:5.[2] IMCUs are often attended by physicians such as general internists or surgeons, with the attending specialty varying across individual units. IMCUs are separate units, often adjacent to the ICU and sharing some equipment, but remain physically separate, with beds dedicated to IMCU care rather than flexibly assigned between ICU and IMCU services. This configuration corresponds to the "dual" model described by Wunsch et al.[3], in contrast to the "integrated" model, where ICU and IMCU beds are flexibly assigned within the same unit.

The Japanese medical reimbursement codes used to define the ICU and IMCU are listed in Supplementary Table 1. Neonatal or obstetric ICUs were not included in this study.

Supplemental Reference

1. Marshall JC, Bosco L, Adhikari NK, et al: What is an intensive care unit? A report of the task force of the World Federation of Societies of Intensive and Critical Care Medicine. J Crit Care 2017;37:270–276. https://doi.org/10.1016/j.jcrc.2016.07.015

2. Case AS, Hochberg CH, Hager DN. The role of intermediate care in supporting critically ill patients and critical care infrastructure. Crit Care Clin. 2024;40:507–22. https://doi.org/10.1016/j.ccc.2024.03.005

3. Wunsch H, Harrison DA, Jones A, Rowan K. The impact of the organization of high-dependency care on acute hospital mortality and patient flow for critically ill patients. Am J Respir Crit Care Med. 2015;191:186–93. https://doi.org/10.1164/rccm.201408-1525OC

Supplementary Table 1. Japanese Medical Reimbursement Codes Used To Define the ICU and IMCU in 2022

Type	Code	Description	Cost per day, JPY	Intensivist	Non-intensivist	Night-shift physician	Nurse to patient ratio	ICU Nurse	Clinical Engineer ****	Reimbursable Duration, days
ICU	A3011	ICU management fee 1	142,110**	≥2 in ICU 24/7	–	Dedicated to ICU	1:2	Required ***	24/7 in hospital	14
ICU	A3012	ICU management fee 2	142,110**	≥2 in ICU 24/7	–	Dedicated to ICU	1:2	Required ***	24/7 in hospital	14 *****
ICU	A3013	ICU management fee 3	96,970**	–	≥1 in ICU 24/7	Dedicated to ICU	1:2	–	–	14
ICU	A3014	ICU management fee 4	96,970**	–	≥1 in ICU 24/7	Dedicated to ICU	1:2	–	–	14 *****
ICU	A3002	Emergency and critical care unit management fee 2	118,020*	–	≥1 in ICU 24/7	Dedicated to ICU	1:2	–	–	14
ICU	A3004	Emergency and critical care unit management fee 4	118,020*	–	≥1 in ICU 24/7	Dedicated to ICU	1:2	–	–	14 *****
ICU	A301-4	Pediatric ICU management fee	163,170**	≥2 in ICU 24/7	–	Dedicated to ICU	1:2	–	–	14 ******
IMCU	A3001	Emergency and critical care unit management fee 1	102,230*	–	≥1 in IMCU 24/7	Dedicated to IMCU	1:4	–	–	14
IMCU	A3003	Emergency and critical care unit management fee 3	102,230*	–	≥1 in IMCU 24/7	Dedicated to IMCU	1:4	–	–	14 *****
IMCU	A301-21	High-care unit management fee 1	68,550	–	≥1 in hospital 24/7	On-call permit	1:4	–	–	21
IMCU	A301-22	High-care unit management fee 2	42,240	–	≥1 in hospital 24/7	On-call permit	1:5	–	–	21
IMCU	A301-3	Stroke-care unit management fee	60,130	–	Neurologist ≥1 in hospital 24/7	On-call permit	1:3	–	–	14

*Cost per day for the first 3 days after admission in 2022

**Cost per day for the first 7 days after admission in 2022

***An ICU nurse is defined as a full-time, dedicated nurse with at least five years' experience in caring for critically ill patients, who has completed appropriate training, and is assigned to the ICU for at least 20 hours per week.

****A clinical engineer is defined as a full-time, dedicated allied health professional responsible for the operation and maintenance of life-support equipment, including mechanical ventilators, high-flow nasal cannula systems, extracorporeal membrane oxygenation (ECMO) devices, renal replacement therapy equipment, and related technologies. Clinical engineers work rotational shifts to ensure that one is present at the hospital 24 hours a day.

*****The reimbursable duration is extended to 60 days for extensive burns.

******The maximum reimbursable duration is extended to 21 days for patients requiring acute blood purification (except peritoneal dialysis), high-risk cardiac surgery, hypoplastic left heart syndrome, acute respiratory distress syndrome, or myocarditis/cardiomyopathy; up to 35 days for patients requiring extracorporeal membrane oxygenation; and up to 55 days for neonates undergoing surgery for congenital heart disease.

ICU, intensive care unit; IMCU, intermediate care unit

Costs are shown in JPY; for reference, we used the 2022 average exchange rate (1 USD = 131·43 JPY).

Supplementary Table 2. Characteristics of Secondary Medical Areas Stratified by ICU Availability Categories

Variables	Secondary Medical Areas without ICUs	Secondary Medical Areas with ICUs, categorized by ICU beds per 100,000 population			
		0·1–2·9	3·0–5·9	6·0–8·9	9·0–27·9
Number of Secondary Medical Area	140 (41·8%)	40 (11·9%)	77 (23·0%)	42 (12·5%)	36 (10·7%)
Population, thousand persons					
Median (IQR)	83 (55–137)	434 (262–704)	428 (238–718)	462 (219–890)	424 (260–671)
Total	14,534 (11·6%)	19867 (15·8%)	45610 (36·3%)	28063 (22·4%)	17479 (13·9%)
Population category, n (%)					
19,219–199,999	125 (89·3)	4 (10·0)	17 (22·1)	9 (21·4)	6 (16·7)
200,000–399,999	15 (10·7)	13 (32·5)	19 (24·7)	11 (26·2)	11 (30·6)
400,000–599,999	0 (0·0)	10 (25·0)	17 (22·1)	6 (14·3)	8 (22·2)
600,000–3,758,300	0 (0·0)	13 (32·5)	24 (31·2)	16 (38·1)	11 (30·6)
Land area, km²					
Median (IQR)	984 (569–1414)	723 (380–1246)	758 (255–1413)	660 (377–1436)	832 (375–1391)
Total	172,378 (46·0%)	37,036 (9·9%)	81,408 (21·7%)	46,235 (12·3%)	37,616 (10·0%)
Population density, persons/km²					
Median (IQR)	86 (50–182)	550 (306–1375)	490 (233–2033)	483 (212–1983)	553 (218–1217)
Number of ICU beds					
Median, IQR	0 (0–0)	8 (6–16)	20 (10–34)	30 (15–64)	48 (36–78)
Total	0 (0·0%)	433 (6·2%)	2144 (30·9%)	2073 (29·9%)	2283 (32·9%)
per 100,000 population	0 (0–0)	2·3 (1·8–2·6)	4·6 (3·8–5·3)	7·2 (6·7–7·8)	11·4 (9·8–13·9)
Annual number of ICU patients	0 (0–0)	482 (340–762)	1361 (598–2309)	1761 (843–4130)	2998 (1934–4990)
Median (IQR)					
Region with IMCU beds	57 (40·7%)	38 (95·0%)	70 (90·9%)	39 (92·9%)	35 (97·2%)
Number of IMCU beds					
Median, IQR	0 (0–9)	46 (28–65)	40 (19–89)	66 (25–126)	58 (31–112)
Total	863 (6·0%)	1978 (13·7%)	4857 (33·7%)	3903 (27·1%)	2798 (19·4%)
per 100,000 population	0·0 (0·0–9·8)	10·9 (6·6–13·1)	10·1 (6·7–13·7)	13·5 (8·2–17·0)	14·9 (9·8–19·9)
Number of acute hospital beds					
Median, IQR	399 (246–664)	1667 (1226–2487)	1870 (1250–3189)	2942 (1322–4562)	2890 (1892–3920)
Total	68,571 (11·0%)	77063 (12·4%)	206110 (33·1%)	155999 (25·1%)	114179 (18·4%)
Per 100,000 population	470 (370–582)	396 (323–492)	476 (387–538)	592 (495–646)	677 (586–761)
Number of all hospital beds					
Median, IQR	848 (558–1290)	3020 (2378–4372)	3442 (2033–5811)	4610 (2432–7228)	4356 (3047–6676)
Total	137,293 (12·9%)	139754 (13·1%)	349306 (32·8%)	259817 (24·4%)	179802 (16·9%)
Per 100,000 population	954 (748–1176)	705 (606–931)	814 (669–945)	990 (845–1196)	1056 (818–1256)
Academic hospital	0 (0·0%)	2 (5·0%)	20 (26·0%)	19 (45·2%)	25 (69·4%)
Tertiary emergency hospital	46 (32·9%)	37 (92·5%)	65 (84·4%)	39 (92·9%)	35 (97·2%)

For the "Total" rows, the values represent sums across SMAs; the percentages in parentheses indicate the share of the overall total (e.g., national population, land area, or total beds) accounted for by each SMA group.
SMA = secondary medical area; ICU = intensive care unit; SMD = standardized mean difference; IMCU = intermediate care unit; IQR = interquartile range

Supplementary Table 3. Full Model Specification for In-hospital Mortality among Patients Admitted to ICUs

Variables	Adjusted risk difference (95% CI)	P value
Residential SMA without ICUs	-0·38 (-0·81 to 0·05)	0·08
Age, years	0·14 (0·12 to 0·15)	<0·001
Male	0·36 (0·15 to 0·57)	0·001
Charlson Comorbidity Index	0·28 (0·17 to 0·39)	<0·001
Level of independence		
Independent	Ref.	–
Mild dependent	-0·51 (-1·26 to 0·23)	0·179
Severe dependent	0·42 (-0·74 to 1·59)	0·478
Location before hospitalization		
Home	Ref.	–
Another hospital	1·35 (0·79 to 1·92)	<0·001
Nursing home	3·53 (2·40 to 4·66)	<0·001
Admission classification		
Elective surgery	Ref.	–
Emergency surgery	2·99 (2·50 to 3·47)	<0·001
Non-surgery	10·87 (10·26 to 11·48)	<0·001
Japan Coma Scale at admission		
Alert	Ref.	–
Dizziness	2·21 (1·58 to 2·84)	<0·001
Somnolence	3·94 (3·00 to 4·88)	<0·001
Coma	19·62 (17·35 to 21·90)	<0·001
Main etiologies for admission		
Cancer	3·60 (3·15 to 4·06)	<0·001
Acute coronary syndrome	-4·42 (-4·92 to -3·92)	<0·001
Aortic dissection or aneurysm	2·49 (1·96 to 3·03)	<0·001
Stroke	1·11 (0·30 to 1·92)	0·007
Acute abdominal diseases	2·83 (2·20 to 3·45)	<0·001
Acute heart failure	-2·19 (-2·93 to -1·46)	<0·001
Trauma	-0·12 (-0·80 to 0·56)	0·731
Post cardiac arrest	24·00 (18·58 to 29·42)	<0·001
Sepsis	11·30 (10·26 to 12·34)	<0·001
Pneumonia	9·36 (7·71 to 11·01)	<0·001
Aspiration	3·37 (1·75 to 4·99)	<0·001
CPR on the day of ICU admission	38·72 (34·26 to 43·18)	<0·001
Organ support therapy on the day of ICU admission		
Invasive mechanical ventilation	6·16 (5·06 to 7·27)	<0·001
Intra-aortic balloon pumping	2·85 (1·69 to 4·01)	<0·001
Extracorporeal membrane oxygenation	9·85 (7·68 to 12·02)	<0·001
Impella	12·83 (8·56 to 17·10)	<0·001
Ventricular assist device	6·84 (0·66 to 13·02)	0·03
Dopamine	2·72 (2·03 to 3·41)	<0·001
Dobutamine	3·09 (2·41 to 3·76)	<0·001
Noradrenaline	2·64 (2·21 to 3·07)	<0·001
Adrenaline	4·50 (3·58 to 5·42)	<0·001
Vasopressin	10·76 (9·21 to 12·30)	<0·001
Continuous renal replacement therapy	12·51 (10·82 to 14·21)	<0·001

Pulmonary artery catheter monitoring	-1·16 (-1·83 to -0·49)	0·001
Cardiac output monitoring	-2·03 (-2·65 to -1·42)	<0·001
Targeted temperature management	-21·69 (-25·53 to -17·85)	<0·001
Intracranial pressure monitoring	5·39 (1·73 to 9·04)	0·004

CI, confidence interval; CPR, cardiopulmonary resuscitation; ICU, intensive care unit; SMA = secondary medical area.

Supplementary Table 4. Characteristics of Critically Ill Patients Requiring Organ Support or Advanced Monitoring Stratified by ICU admission status

Variables	Overall N=467,200	Patients without ICU admission N=306,591	Patients with ICU admission N=160,609	SMD %
**Geographic healthcare factors**				
ICU beds per 100,000 population in residential SMA				
0	54,952 (11·8)	41,295 (13·5)	13,657 (8·5)	-0·16
0·1–2·9	63,614 (13·6)	43,898 (14·3)	19,716 (12·3)	-0·06
3·0–5·9	164,300 (35·2)	108,085 (35·3)	56,215 (35·0)	-0·01
6·0–8·9	113,255 (24·2)	72,643 (23·7)	40,612 (25·3)	0·04
9·0–27·9	71,079 (15·2)	40,670 (13·3)	30,409 (18·9)	0·16
ICU beds per 100,000 population in hospital SMA				
0	24,893 (5·3)	24,634 (8·0)	0 (0·0)	–
0·1–2·9	45,331 (9·7)	35,880 (11·7)	9,710 (6·1)	-0·21
3·0–5·9	157,250 (33·7)	105,581 (34·4)	51,669 (32·2)	-0·05
6·0–8·9	129,359 (27·7)	82,461 (26·9)	46,898 (29·2)	0·05
9·0–27·9	110,367 (23·6)	58,035 (18·9)	52,332 (32·6)	0·32
Hospital within the same SMA as residence	339,469 (72·7)	234,946 (76·6)	104,523 (65·1)	-0·26
Distance from residence to hospital, km, mean (SD)	14·2 (46·3)	13·0 (44·3)	16·5 (49·8)	0·07
Distance from residence to hospital, km, median (IQR)	5·8 (2·9–12·4)	5·5 (2·8–11·4)	6·6 (3·2–14·9)	0·07
**Patient characteristics**				
Age, years	68·5 (21·6)	68·7 (23·0)	68·1 (18·6)	-0·03
Male	274,685 (58·8)	175,027 (57·1)	99,658 (62·1)	0·10
Charlson Comorbidity Index	1·1 (1·5)	1·1 (1·5)	1·2 (1·5)	0·06
Level of independence				
Independent	380,112 (81·4)	241,875 (78·9)	138,237 (86·1)	0·19
Mild dependent	48,096 (10·3)	34,394 (11·2)	13,702 (8·5)	-0·09
Severe dependent	38,992 (8·3)	30,322 (9·9)	8,670 (5·4)	-0·17
Location before hospitalization				
Home	410,332 (87·8)	267,949 (87·4)	142,383 (88·7)	0·04
Another hospital	31,600 (6·8)	17,770 (5·8)	13,830 (8·6)	0·11
Nursing home	25,268 (5·4)	20,872 (6·8)	4,396 (2·7)	-0·19
Admission classification				
Elective surgery	128,197 (27·4)	69,469 (22·7)	58,728 (36·6)	0·31
Emergency surgery	71,684 (15·3)	33,111 (10·8)	38,573 (24·0)	0·35
Non-surgery	267,319 (57·2)	204,011 (66·5)	63,308 (39·4)	-0·56
Japan Coma Scale at admission				
Alert	322,214 (69·0)	213,821 (69·7)	108,393 (67·5)	-0·05
Dizziness	56,533 (12·1)	37,540 (12·2)	18,993 (11·8)	-0·01
Somnolence	19,185 (4·1)	11,806 (3·9)	7,379 (4·6)	0·04
Coma	69,268 (14·8)	43,424 (14·2)	25,844 (16·1)	0·05
Main etiologies for admission				
Cancer	69,023 (14·8)	43,641 (14·2)	25,382 (15·8)	0·04
Acute coronary syndrome	47,490 (10·2)	27,805 (9·1)	19,685 (12·3)	0·10
Aortic dissection or aneurysm	26,481 (5·7)	9,523 (3·1)	16,958 (10·6)	0·30
Stroke	23,083 (4·9)	12,617 (4·1)	10,466 (6·5)	0·11
Acute abdominal diseases	33,348 (7·1)	21,972 (7·2)	11,376 (7·1)	0·00
Acute heart failure	43,215 (9·2)	32,418 (10·6)	10,797 (6·7)	-0·14

Trauma	20,624 (4·4)	14,186 (4·6)	6,438 (4·0)	-0·03
Post cardiac arrest	29,140 (6·2)	19,443 (6·3)	9,697 (6·0)	-0·01
Sepsis	23,191 (5·0)	14,506 (4·7)	8,685 (5·4)	0·03
Pneumonia	9,456 (2·0)	7,271 (2·4)	2,185 (1·4)	-0·07
Aspiration	10,298 (2·2)	7,928 (2·6)	2,370 (1·5)	-0·08
CPR on the start day of organ support or advanced monitoring	45,164 (9·7)	32,333 (10·5)	12,831 (8·0)	-0·09
Organ support therapy on the start day of organ support or advanced monitoring				
Invasive mechanical ventilation	162,621 (34·8)	101,635 (33·2)	60,986 (38·0)	0·10
Intra-aortic balloon pumping	6,327 (1·4)	1,813 (0·6)	4,514 (2·8)	0·17
Extracorporeal membrane oxygenation	3,163 (0·7)	658 (0·2)	2,505 (1·6)	0·14
Impella	1,110 (0·2)	162 (0·1)	948 (0·6)	0·10
Ventricular assist device	577 (0·1)	431 (0·1)	146 (0·1)	-0·01
Dopamine	79,741 (17·1)	52,758 (17·2)	26,983 (16·8)	-0·01
Dobutamine	60,163 (12·9)	28,406 (9·3)	31,757 (19·8)	0·30
Noradrenaline	240,040 (51·4)	144,231 (47·0)	95,809 (59·7)	0·25
Adrenaline	65,574 (14·0)	41,236 (13·4)	24,338 (15·2)	0·05
Vasopressin	15,630 (3·3)	7,443 (2·4)	8,187 (5·1)	0·14
Continuous renal replacement therapy	8,879 (1·9)	2,446 (0·8)	6,433 (4·0)	0·21
Pulmonary artery catheter monitoring	22,011 (4·7)	3,794 (1·2)	18,217 (11·3)	0·43
Cardiac output monitoring	35,591 (7·6)	10,339 (3·4)	25,252 (15·7)	0·43
Targeted temperature management	2,748 (0·6)	712 (0·2)	2,036 (1·3)	0·12
Intracranial pressure monitoring	810 (0·2)	253 (0·1)	557 (0·3)	0·06
**Outcomes**				
ICU admission	160,609 (34·4)	0 (0·0)	160,609 (100·0)	–
In-hospital mortality	108,343 (23·2)	77,499 (25·3)	30,844 (19·2)	-0·15
IMCU admission	140,303 (30·0)	110,868 (36·2)	29,435 (18·3)	-0·41
No ICU/IMCU admission	195,723 (41·9)	195,723 (63·8)	0 (0·0)	–
Length of hospital stay, days	16·0 (7·0–31·0)	13·0 (5·0–28·0)	21·0 (12·0–37·0)	0·05
Hospitalization costs, million yen	1·9 (0·9–3·5)	1·4 (0·7–2·4)	3·3 (1·8–5·5)	0·45
Early hospital transfer	2,474 (0·5)	1,884 (0·6)	590 (0·4)	-0·04

Baseline characteristics were assessed at hospital admission. Cardiopulmonary resuscitation and organ support therapy were assessed on the day of initiation of organ support or advanced monitoring.
SMD indicates the magnitude of between-group imbalance; values >0·10 are commonly considered meaningful.
Costs are shown in JPY; for reference, we used the 2022 average exchange rate (1 USD = 131·43 JPY).
SMA = secondary medical area; ICU = intensive care unit; IMCU = inter-mediate care unit; SMD = standardized mean difference; SD = standard deviation; IQR = interquartile range; CPR = cardiopulmonary resuscitation

Supplementary Table 5. Full Model Specification for ICU Admission among Critically Ill Patients Requiring Organ Support or Advanced Monitoring

Variables	Adjusted risk difference (95% CI)	P value
Residential SMA without ICUs	-11·13 (-14·07 to -8·20)	<0·001
Age, years	0·01 (-0·03 to 0·04)	0·715
Male	2·22 (1·78 to 2·67)	<0·001
Charlson Comorbidity Index	0·56 (0·25 to 0·86)	<0·001
Level of independence		
Independent	Ref.	–
Mild dependent	-3·54 (-5·05 to -2·04)	<0·001
Severe dependent	-6·35 (-8·75 to -3·96)	<0·001
Location before hospitalization		
Home	Ref.	–
Another hospital	6·11 (4·74 to 7·47)	<0·001
Nursing home	-6·60 (-8·04 to -5·15)	<0·001
Admission classification		
Elective surgery	Ref.	–
Emergency surgery	2·04 (0·06 to 4·01)	0·043
Non-surgery	-24·68 (-27·31 to -22·05)	<0·001
Japan Coma Scale at admission		
Alert	Ref.	–
Dizziness	7·67 (6·05 to 9·29)	<0·001
Somnolence	11·16 (9·39 to 12·93)	<0·001
Coma	11·44 (8·97 to 13·90)	<0·001
Main etiologies for admission		
Cancer	3·62 (1·16 to 6·08)	0·004
Acute coronary syndrome	10·75 (8·98 to 12·52)	<0·001
Aortic dissection or aneurysm	20·09 (18·04 to 22·14)	<0·001
Stroke	7·34 (4·40 to 10·29)	<0·001
Acute abdominal diseases	0·53 (-1·05 to 2·12)	0·509
Acute heart failure	3·45 (1·76 to 5·14)	<0·001
Trauma	-3·30 (-5·66 to -0·93)	0·006
Post cardiac arrest	7·64 (3·49 to 11·79)	<0·001
Sepsis	8·05 (6·74 to 9·35)	<0·001
Pneumonia	0·67 (-0·67 to 2·02)	0·328
Aspiration	0·86 (-0·61 to 2·33)	0·25
CPR on the start day of organ support or advanced monitoring	-14·20 (-16·64 to -11·75)	<0·001
Organ support therapy on the start day of organ support or advanced monitoring		
Invasive mechanical ventilation	21·40 (19·85 to 22·96)	<0·001
Intra-aortic balloon pumping	29·18 (26·34 to 32·02)	<0·001
Extracorporeal membrane oxygenation	21·02 (17·66 to 24·38)	<0·001
Impella	16·41 (9·81 to 23·02)	<0·001
Ventricular assist device	16·55 (10·89 to 22·21)	<0·001
Dopamine	1·93 (-0·83 to 4·69)	0·17
Dobutamine	14·74 (12·80 to 16·67)	<0·001
Noradrenaline	12·64 (10·98 to 14·31)	<0·001
Adrenaline	3·89 (1·81 to 5·96)	<0·001
Vasopressin	9·71 (7·74 to 11·67)	<0·001

Continuous renal replacement therapy	36·26 (33·68 to 38·85)	<0·001
Pulmonary artery catheter monitoring	19·95 (15·71 to 24·19)	<0·001
Cardiac output monitoring	20·19 (16·46 to 23·93)	<0·001
Targeted temperature management	20·85 (16·79 to 24·90)	<0·001
Intracranial pressure monitoring	20·01 (11·03 to 28·99)	<0·001

CI, confidence interval; CPR, cardiopulmonary resuscitation; ICU, intensive care unit; SMA = secondary medical area.

Supplementary Table 6. Full Model Specification for In-hospital Mortality among Critically Ill Patients Requiring Organ Support or Advanced Monitoring

Variables	Adjusted risk difference (95% CI)	P value
Residential SMA without ICUs	0·96 (0·17 to 1·75)	0·017
Age, years	0·26 (0·25 to 0·27)	<0·001
Male	1·14 (0·91 to 1·36)	<0·001
Charlson Comorbidity Index	0·84 (0·73 to 0·95)	<0·001
Level of independence		
Independent	Ref.	–
Mild dependent	2·87 (2·04 to 3·70)	<0·001
Severe dependent	6·47 (5·42 to 7·53)	<0·001
Location before hospitalization		
Home	Ref.	–
Another hospital	3·98 (3·35 to 4·61)	<0·001
Nursing home	5·14 (4·38 to 5·90)	<0·001
Admission classification		
Elective surgery	Ref.	–
Emergency surgery	4·16 (3·62 to 4·70)	<0·001
Non-surgery	21·37 (20·41 to 22·33)	<0·001
Japan Coma Scale at admission		
Alert	Ref.	–
Dizziness	6·12 (5·40 to 6·84)	<0·001
Somnolence	7·80 (6·88 to 8·72)	<0·001
Coma	20·76 (19·71 to 21·80)	<0·001
Main etiologies for admission		
Cancer	9·09 (8·39 to 9·79)	<0·001
Acute coronary syndrome	-10·12 (-10·89 to -9·36)	<0·001
Aortic dissection or aneurysm	3·20 (2·55 to 3·85)	<0·001
Stroke	5·04 (4·17 to 5·90)	<0·001
Acute abdominal diseases	5·21 (4·58 to 5·85)	<0·001
Acute heart failure	-6·67 (-7·42 to -5·92)	<0·001
Trauma	0·30 (-0·49 to 1·08)	0·457
Post cardiac arrest	10·15 (9·02 to 11·28)	<0·001
Sepsis	3·70 (2·76 to 4·64)	<0·001
Pneumonia	13·41 (12·19 to 14·62)	<0·001
Aspiration	9·64 (8·40 to 10·88)	<0·001
CPR on the start day of organ support or advanced monitoring	36·18 (35·04 to 37·32)	<0·001
Organ support therapy on the start day of organ support or advanced monitoring		
Invasive mechanical ventilation	6·49 (5·89 to 7·10)	<0·001
Intra-aortic balloon pumping	1·71 (0·73 to 2·68)	0·001
Extracorporeal membrane oxygenation	8·56 (6·78 to 10·34)	<0·001
Impella	11·33 (7·54 to 15·12)	<0·001
Ventricular assist device	-1·51 (-4·33 to 1·31)	0·295
Dopamine	6·63 (5·77 to 7·48)	<0·001
Dobutamine	4·67 (4·05 to 5·29)	<0·001
Noradrenaline	1·00 (0·48 to 1·52)	<0·001
Adrenaline	13·16 (12·14 to 14·18)	<0·001
Vasopressin	10·18 (9·04 to 11·32)	<0·001

Continuous renal replacement therapy	8·14 (6·79 to 9·49)	<0·001
Pulmonary artery catheter monitoring	0·66 (-0·50 to 1·83)	0·265
Cardiac output monitoring	-2·17 (-2·90 to -1·45)	<0·001
Targeted temperature management	-17·35 (-19·63 to -15·07)	<0·001
Intracranial pressure monitoring	0·78 (-2·36 to 3·92)	0·626

CI, confidence interval; CPR, cardiopulmonary resuscitation; ICU, intensive care unit; SMA = secondary medical area.

Supplementary Table 7. Results of the Sensitivity and Subgroup Analyses in Patients Admitted to ICUs

Outcome	Values	Unadjusted risk difference (95% CI)	P value	Adjusted risk difference (95% CI)	P value
<u>**In-hospital mortality, %**</u>					
**Main Analysis**					
SMAs without ICUs	1,868/21,434 (8·7)	-4·42 (-5·58 to -3·27)	<0·001	-0·38 (-0·81 to 0·05)	0·080
SMAs with ICUs	34,349/261,460 (13·1)	Ref.	–	Ref.	–
**Sensitivity Analysis 1· Stratified by Residential ICU Availability Categories**					
ICU beds per 100,000 population					
0	1,868/21,434 (8·7)	Ref.	–	Ref.	–
0·1–2·9	3,786/33,439 (11·3)	2·61 (0·07 to 5·15)	0·044	0·28 (–0·35 to 0·92)	0·384
3·0–5·9	13,928/99,355 (14·0)	5·30 (3·52 to 7·09)	<0·001	0·54 (–0·02 to 1·10)	0·059
6·0–8·9	9,212/72,796 (12·7)	3·94 (2·42 to 5·46)	<0·001	0·28 (–0·23 to 0·79)	0·282
9·0–27·9	7,423/55,870 (13·3)	4·57 (2·67 to 6·47)	<0·001	0·30 (–0·57 to 1·16)	0·500
**Sensitivity Analysis 2· Stratified by SMA population size**					
Population category, n (%)					
19,219–199,999					
SMAs without ICUs	1,464/16,879 (8·7)	-3·70 (-6·03 to -1·37)	0·002	-0·61 (-1·52 to 0·31)	0·194
SMAs with ICUs	1,614/13,047 (12·4)	Ref.	–	Ref.	–
200,000–399,999					
SMAs without ICUs	404/4,555 (8·9)	-4·56 (-7·11 to -2·00)	<0·001	-0·29 (-1·10 to 0·52)	0·486
SMAs with ICUs	4,833/35,995 (13·4)	Ref.	–	Ref.	–
**Subgroup analysis 1· Stratified by Admission Classification**					
Elective surgery					
SMAs without ICUs	138/10,914 (1·3)	0·07 (-0·18 to 0·31)	0·584	-0·21 (-0·45 to 0·03)	0·092
SMAs with ICUs	1,229/102,753 (1·2)	Ref.	–	Ref.	–
Emergency surgery/Non-surgery					
SMAs without ICUs	1,730/10,520 (16·4)	-4·42 (-5·93 to -2·92)	<0·001	-0·47 (-1·25 to 0·32)	0·243
SMAs with ICUs	33,120/158,707 (20·9)	Ref.	–	Ref.	–
**Subgroup analysis 2· Stratified by Organ Support Therapy**					
Invasive mechanical ventilation					
SMAs without ICUs	910/3,426 (26·6)	-12·38 (-15·98 to -8·78)	<0·001	0·38 (-1·13 to 1·90)	0·622
SMAs with ICUs	18,687/47,988 (38·9)	Ref.	–	Ref.	–
Mechanical circulatory support					
SMAs without ICUs	157/626 (25·1)	-8·03 (-12·31 to -3·75)	<0·001	-2·09 (-5·55 to 1·37)	0·237
SMAs with ICUs	2,367/7,149 (33·1)	Ref.	–	Ref.	–
Continuous renal replacement therapy					
SMAs without ICUs	219/533 (41·1)	2·29 (-1·96 to 6·53)	0·291	1·98 (-2·10 to 6·05)	0·342
SMAs with ICUs	2,193/5,652 (38·8)	Ref.	–	Ref.	–
Vasopressors					
SMAs without ICUs	1,121/10,597 (10·6)	-5·18 (-6·48 to -3·88)	<0·001	-0·92 (-2·01 to 0·23)	0·109
SMAs with ICUs	15,702/99,642 (15·8)	Ref.	–	Ref.	–
Monitoring					
SMAs without ICUs	307/4,062 (7·6)	-1·98 (-3·33 to -0·64)	0·004	0·43 (-0·48 to 1·34)	0·355
SMAs with ICUs	2,859/29,962 (9·5)	Ref.	–	Ref.	–

The models were adjusted for age, sex, Charlson Comorbidity Index, level of independence, location before hospitalization, admission classification, level of consciousness, main etiologies for admission, cardiopulmonary resuscitation on the day of ICU admission, and organ support therapy on the day of ICU admission.

Risk differences were defined as (SMAs without ICUs − SMAs with ICUs), with SMAs with ICUs as the reference category. Positive values indicate higher risk in SMAs without ICUs, and negative values indicate lower risk. SMA = secondary medical area; ICU = intensive care unit; CI = confidence interval

Supplementary Table 8. Results of the Sensitivity and Subgroup Analyses in Critically Ill Patients Requiring Organ Support or Advanced Monitoring

Outcome	Values	Unadjusted risk difference (95% CI)	P value	Adjusted risk difference (95% CI)	P value
ICU admission, %					
**Main Analysis**					
SMAs without ICUs	13,657/54,952 (24·9)	-10·79 (-14·59 to -7·00)	<0·001	-11·13 (-14·07 to -8·20)	<0·001
SMAs with ICUs	146,952/412,248 (35·6)	Ref.	–	Ref.	–
**Sensitivity Analysis 1. Stratified by Residential ICU Availability Categories**					
ICU beds per 100,000 population					
0	13,657/54,952 (24·9)	Ref.	–	Ref.	–
0·1–2·9	19,716/63,614 (31·0)	6·55 (2·43 to 10·7)	0·002	6·55 (2·43 to 10·7)	0·002
3·0–5·9	56,215/164,300 (34·2)	9·92 (6·46 to 13·39)	<0·001	9·92 (6·46 to 13·39)	<0·001
6·0–8·9	40,612/113,255 (35·9)	11·44 (7·82 to 15·05)	<0·001	11·44 (7·82 to 15·05)	<0·001
9·0–27·9	30,409/71,079 (42·8)	17·58 (13·67 to 21·49)	<0·001	17·58 (13·67 to 21·49)	<0·001
**Sensitivity Analysis 2. Stratified by SMA population size**					
Population category, n (%)					
19,219–199,999					
SMAs without ICUs	10,807/42,913 (25·2)	-2·53 (-7·48 to 2·42)	0·316	-5·04 (-9·06 to -1·03)	0·014
SMAs with ICUs	8,288/29,903 (27·7)	Ref.	–	Ref.	–
200,000–399,999					
SMAs without ICUs	2,850/12,039 (23·7)	-9·44 (-18·12 to -0·75)	0·033	-9·84 (-16·66 to -3·03)	0·005
SMAs with ICUs	19,649/59,345 (33·1)	Ref.	–	Ref.	–
**Sensitivity Analysis 3. Excluding receipt of organ support therapy from the adjustment set**					
SMAs without ICUs	13,657/54,952 (24·9)	–	–	-11·26 (-14·51 to -8·01)	<0·001
SMAs with ICUs	146,952/412,248 (35·6)	–	–	Ref.	–
**Sensitivity Analysis 4. Excluding patients with dopamine-only, dobutamine-only, or advanced-monitoring-only**					
SMAs without ICUs	12,245/44,192 (27·7)	-10·36 (-14·46 to -6·25)	<0·001	-11·90 (-15·10 to -8·69)	<0·001
SMAs with ICUs	132,801/348,887 (38·1)	Ref.	–	Ref.	–
**Subgroup analysis 1. Stratified by Admission Classification**					
Elective surgery					
SMAs without ICUs	6,628/16,502 (40·2)	-6·48 (-11·80 to -1·16)	0·017	-8·43 (-12·64 to -4·22)	<0·001
SMAs with ICUs	52,100/111,695 (46·6)	Ref.	–	Ref.	–
Emergency surgery/Non-surgery					
SMAs without ICUs	7,029/38,450 (18·3)	-13·28 (-16·71 to -9·84)	<0·001	-12·37 (-15·11 to -9·63)	<0·001
SMAs with ICUs	94,852/300,553 (31·6)	Ref.	–	Ref.	–
**Subgroup analysis 2. Stratified by Organ Support Therapy**					
Invasive mechanical ventilation					
SMAs without ICUs	4,042/17,201 (23·5)	-15·66 (-20·65 to -10·67)	<0·001	-16·37 (-19·98 to -12·76)	<0·001
SMAs with ICUs	56,944/145,420 (39·2)	Ref.	–	Ref.	–
Mechanical circulatory support					
SMAs without ICUs	643/1,196 (53·8)	-20·78 (-29·55 to -12·00)	<0·001	-19·47 (-27·43 to -11·51)	<0·001
SMAs with ICUs	7,243/9,717 (74·5)	Ref.	–	Ref.	–
Continuous renal replacement therapy					
SMAs without ICUs	542/994 (54·5)	-20·18 (-28·28 to -12·09)	<0·001	-19·85 (-26·87 to -12·82)	<0·001
SMAs with ICUs	5,891/7,885 (74·7)	Ref.	–	Ref.	–
Vasopressors					
SMAs without ICUs	11,257/43,565 (25·8)	-9·80 (-13·76 to -5·85)	<0·001	-10·00 (-12·96 to -7·05)	<0·001

SMAs with ICUs	113,732/319,098 (35·6)	Ref.	–	Ref.	–
Monitoring					
SMAs without ICUs	4,167/6,213 (67·1)	-5·39 (-11·33 to 0·56)	0·076	-8·31 (-13·10 to -3·51)	0·001
SMAs with ICUs	31,251/43,131 (72·5)	Ref.	–	Ref.	–
<u>**In-hospital mortality, %**</u>					
**Main Analysis**					
SMAs without ICUs	12,467/54,952 (22·7)	-0·57 (-2·74 to 1·61)	0·608	0·96 (0·17 to 1·75)	0·017
SMAs with ICUs	95,876/412,248 (23·3)	Ref.	–	Ref.	–
**Sensitivity Analysis 1. Stratified by Residential ICU Availability Categories**					
ICU beds per 100,000 population in residential SMA					
0	12,467/54,952 (22·7)	Ref.	–	Ref.	–
0·1–2·9	14,838/63,614 (23·3)	0·24 (-0·84 to 1·31)	0·666	0·24 (-0·84 to 1·31)	0·666
3·0–5·9	39,507/164,300 (24·0)	-0·81 (-1·70 to 0·08)	0·073	-0·81 (-1·70 to 0·08)	0·073
6·0–8·9	25,804/113,255 (22·8)	-1·32 (-2·23 to -0·41)	0·004	-1·32 (-2·23 to -0·41)	0·004
9·0–27·9	15,727/71,079 (22·1)	-1·83 (-2·90 to -0·76)	0·001	-1·83 (-2·90 to -0·76)	0·001
**Sensitivity Analysis 2. Stratified by SMA population size**					
Population category, n (%)					
19,219–199,999					
SMAs without ICUs	9,643/42,913 (22·5)	-0·12 (-3·30 to 3·06)	0·942	1·04 (-0·07 to 2·15)	0·065
SMAs with ICUs	6,755/29,903 (22·6)	Ref.	–	Ref.	–
200,000–399,999					
SMAs without ICUs	2,824/12,039 (23·5)	-1·11 (-5·64 to 3·41)	0·630	1·85 (0·19 to 3·51)	0·029
SMAs with ICUs	14,581/59,345 (24·6)	Ref.	–	Ref.	–
**Sensitivity Analysis 3. Excluding receipt of organ support therapy from the adjustment set**					
SMAs without ICUs	12,467/54,952 (22·7)	–	–	1·49 (0·49 to 2·49)	0·003
SMAs with ICUs	95,876/412,248 (23·3)	–	–	Ref.	–
**Sensitivity Analysis 4. Excluding patients with dopamine-only, dobutamine-only, or advanced-monitoring-only**					
SMAs without ICUs	10,493/44,192 (23·7)	-0·94 (-3·38 to 1·49)	0·448	0·92 (0·12 to 17·1)	0·023
SMAs with ICUs	86,127/348,887 (24·7)	Ref.	–	Ref.	–
**Subgroup analysis 1. Stratified by Admission Classification**					
Elective surgery					
SMAs without ICUs	351/16,502 (2·1)	0·07 (-0·26 to 0·40)	0·688	-0·15 (-0·44 to 0·14)	0·305
SMAs with ICUs	2,300/111,695 (2·1)	Ref.	–	Ref.	–
Emergency surgery/Non-surgery					
SMAs without ICUs	12,116/38,450 (31·5)	0·38 (-1·87 to 2·62)	0·742	1·47 (0·49 to 2·45)	0·003
SMAs with ICUs	93,576/300,553 (31·1)	Ref.	–	Ref.	–
**Subgroup analysis 2. Stratified by Organ Support Therapy**					
Invasive mechanical ventilation					
SMAs without ICUs	7,387/17,201 (42·9)	0·19 (-2·96 to 3·35)	0·904	2·89 (1·88 to 3·90)	<0·001
SMAs with ICUs	62,169/145,420 (42·8)	Ref.	–	Ref.	–
Mechanical circulatory support					
SMAs without ICUs	282/1,196 (23·6)	-7·96 (-10·99 to -4·94)	<0·001	-1·23 (-3·55 to 1·09)	0·299
SMAs with ICUs	3,065/9,717 (31·5)	Ref.	–	Ref.	–
Continuous renal replacement therapy					
SMAs without ICUs	372/994 (37·4)	1·37 (-2·33 to 5·07)	0·469	1·73 (-1·41 to 4·87)	0·281
SMAs with ICUs	2,843/7,885 (36·1)	Ref.	–	Ref.	–
Vasopressors					
SMAs without ICUs	9,774/43,565 (22·4)	-1·88 (-4·27 to 0·51)	0·124	0·49 (-0·34 to 1·31)	0·246
SMAs with ICUs	77,580/319,098 (24·3)	Ref.	–	Ref.	–

Monitoring					
SMAs without ICUs	430/6,213 (6·9)	-2·07 (-3·20 to -0·94)	<0·001	0·20 (-0·54 to 0·94)	0·601
SMAs with ICUs	3,878/43,131 (9·0)	Ref.	–	Ref.	–

The models were adjusted for age, sex, Charlson Comorbidity Index, level of independence, location before hospitalization, admission classification, level of consciousness, main etiologies for admission, cardiopulmonary resuscitation on the day of ICU admission, and organ support therapy on the day of initiation of organ support or advanced monitoring.
The models in the sensitivity analysis 3 "Excluding receipt of organ support therapy from the adjustment set" were adjusted for age, sex, Charlson Comorbidity Index, level of independence, location before hospitalization, admission classification, level of consciousness, and main etiologies for admission.
Risk differences were defined as (SMAs without ICUs − SMAs with ICUs), with SMAs with ICUs as the reference category. Positive values indicate higher risk in SMAs without ICUs, and negative values indicate lower risk.
SMA = secondary medical area; ICU = intensive care unit; CI = confidence interval

Supplementary Figure 1. Exposure Assignment and Subsequent Care Pathways Captured in This Study

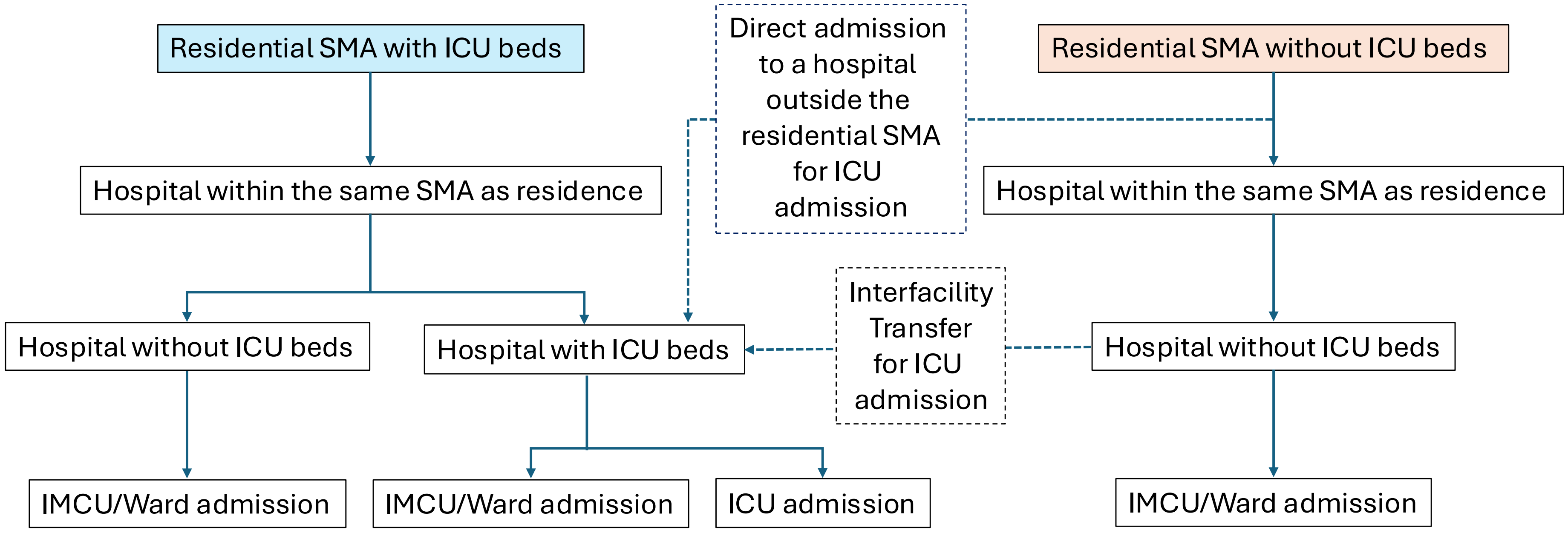

Exposure was defined at the residential SMA level (blue: with ICU-designated beds; orange: without ICU-designated beds).
Dashed lines indicate a potential pathway for ICU admission at the residential SMA without ICU beds, i.e., direct admission to an ICU-equipped hospital outside the residential SMA or interfacility transfer when ICU admission is pursued.
SMA = secondary medical area; ICU = intensive care unit; IMCU = intermediate care unit;

Supplementary Figure 2. Relationship Between the Population Size of the Secondary Medical Area and ICU Bed Density

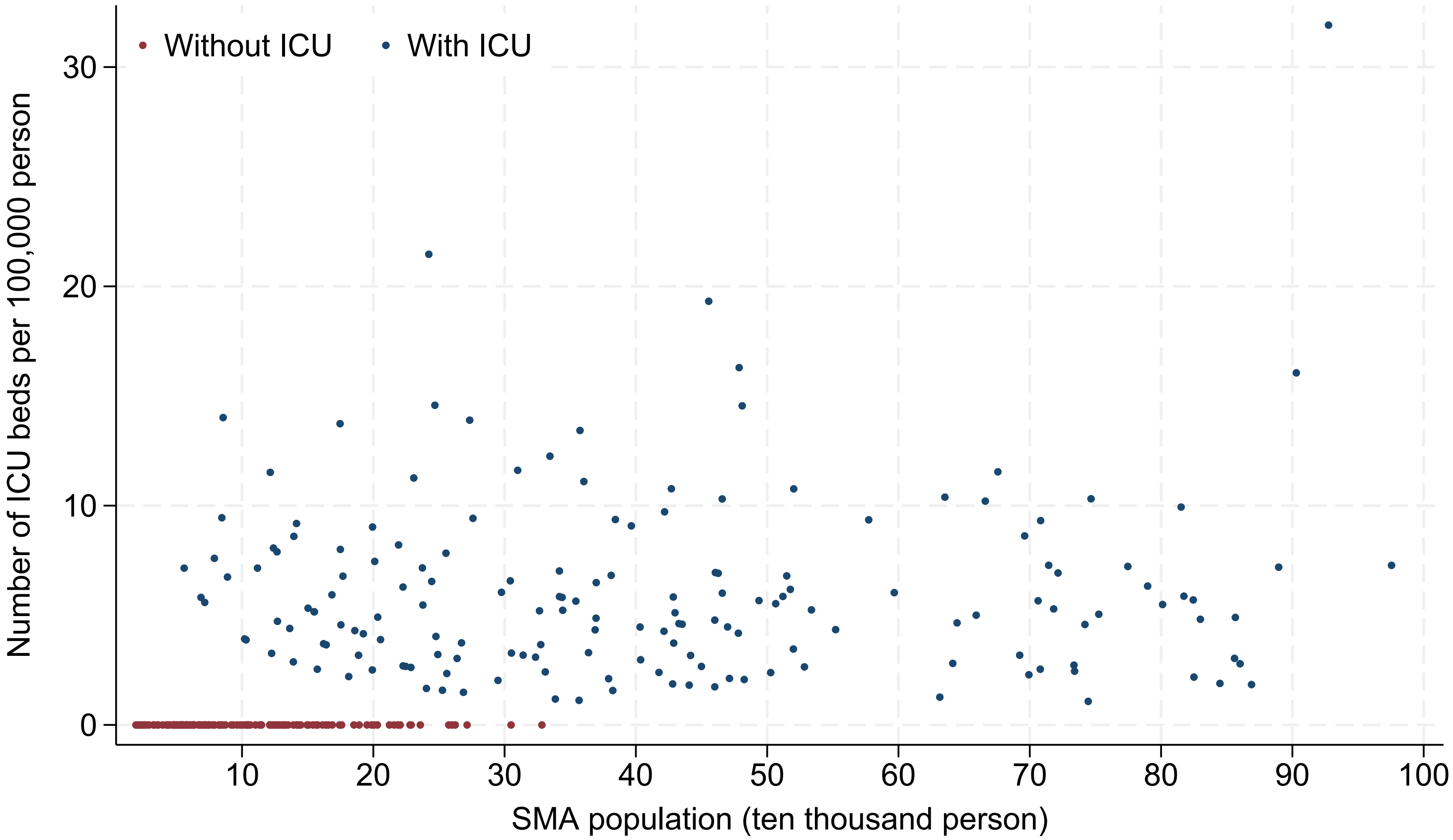

Each point represents an SMA. The x-axis shows the population of the SMA (in units of 10,000 persons), and the y-axis shows the number of ICU beds per 100,000 population. SMAs are colored by ICU availability (without ICU beds vs with ≥1 ICU bed). For visualization, SMAs with populations >100 (i.e., >1,000,000 persons) are not shown; all such SMAs had ≥1 ICU bed.

ICU = intensive care unit; SMA: secondary medical area

Supplementary Figure 3. Geographic Distribution of ICU Beds (A3011/A3012) per 100,000 Population Across Secondary Medical Areas in Japan

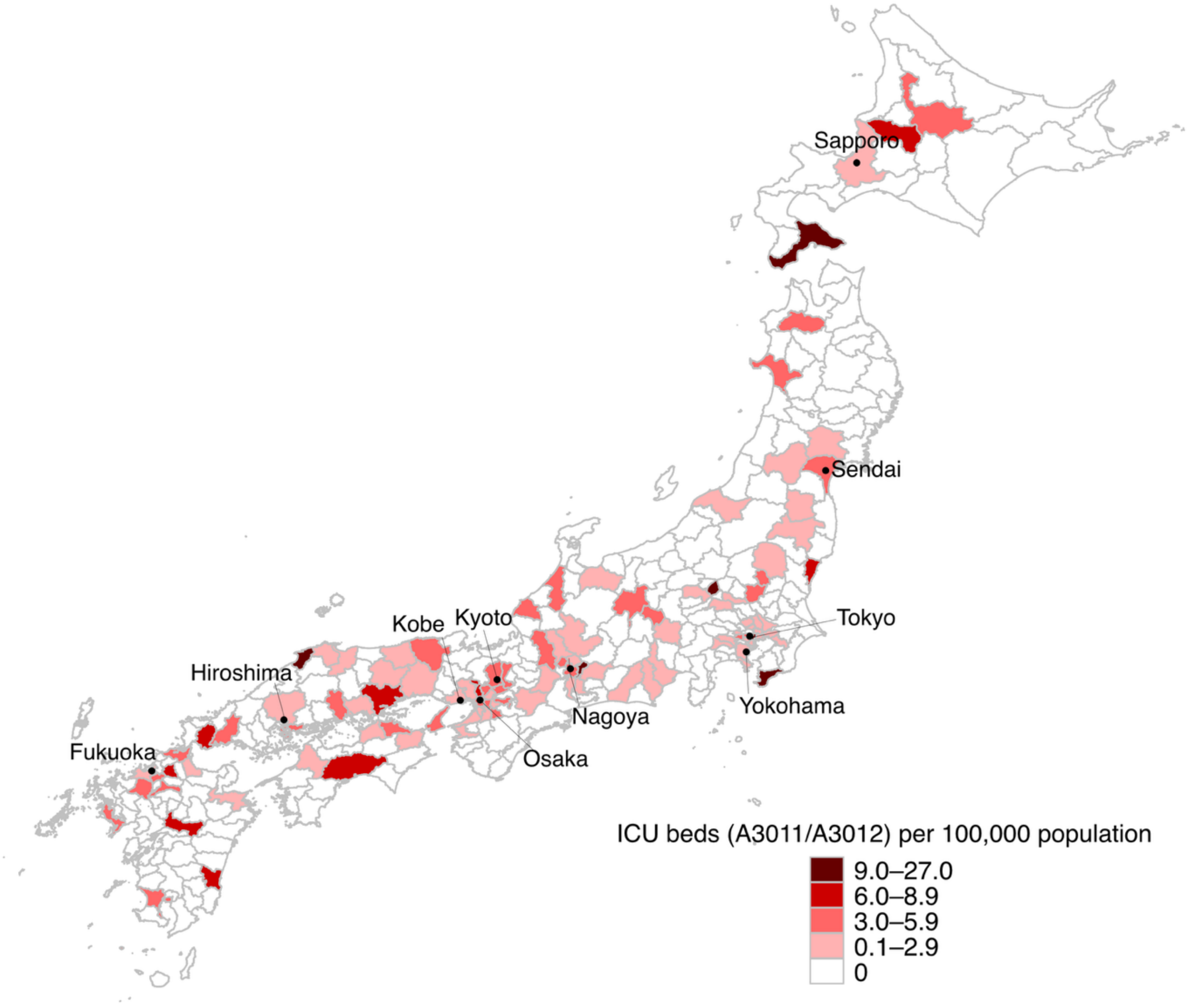

Secondary medical areas without ICU beds (A3011/A3012) are shown in white. The color intensity reflects ICU bed (A3011/A3012) density per 100,000 population.
ICU = intensive care unit

Supplementary Figure 4. Geographic Distribution of ICU Beds (A3013/A3014) per 100,000 Population Across Secondary Medical Areas in Japan

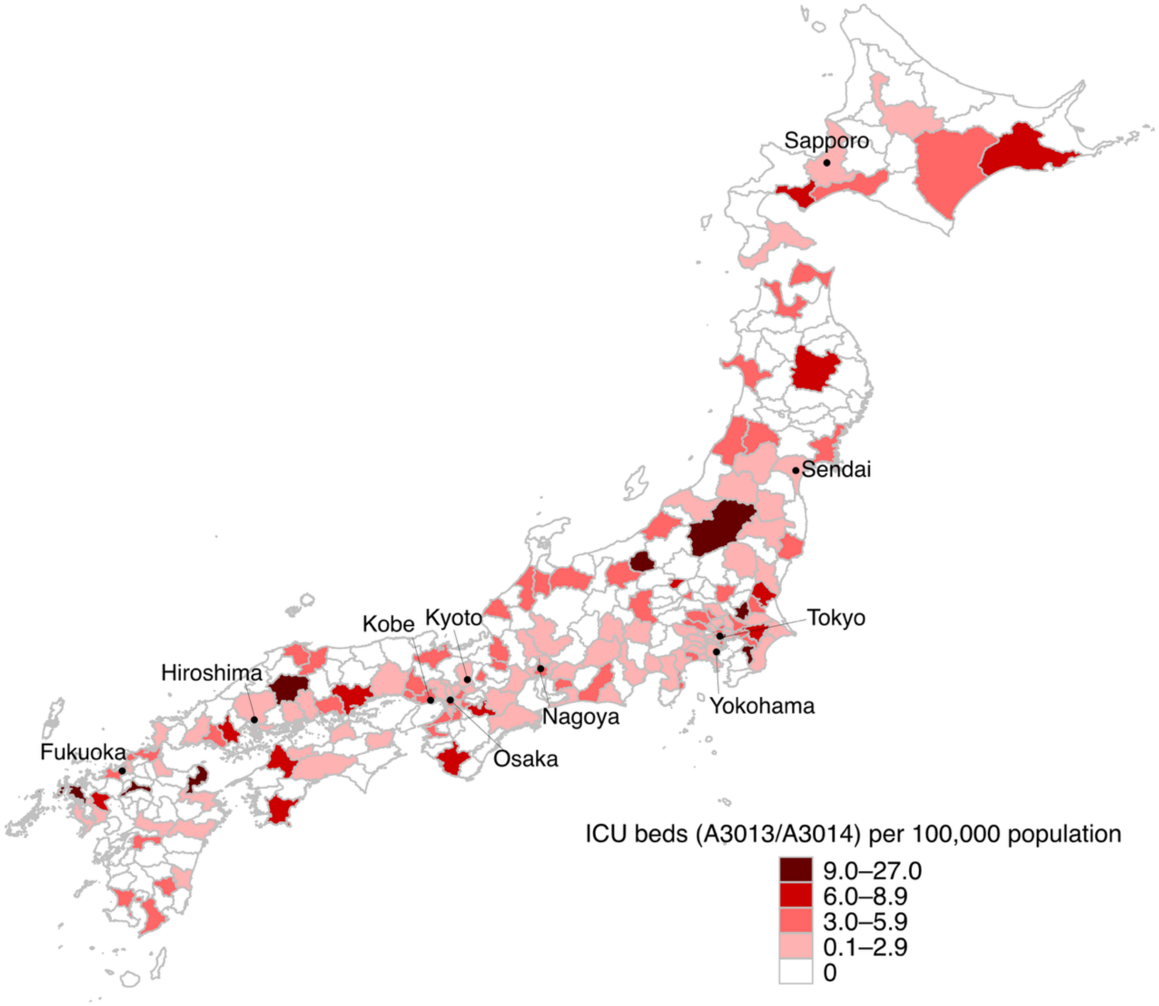

Secondary medical areas without ICU beds (A3013/A3014) are shown in white. The color intensity reflects ICU bed (A3013/A3014) density per 100,000 population.
ICU = intensive care unit

Supplementary Figure 5. Geographic Distribution of ICU Beds (A3002/A3004) per 100,000 Population Across Secondary Medical Areas in Japan

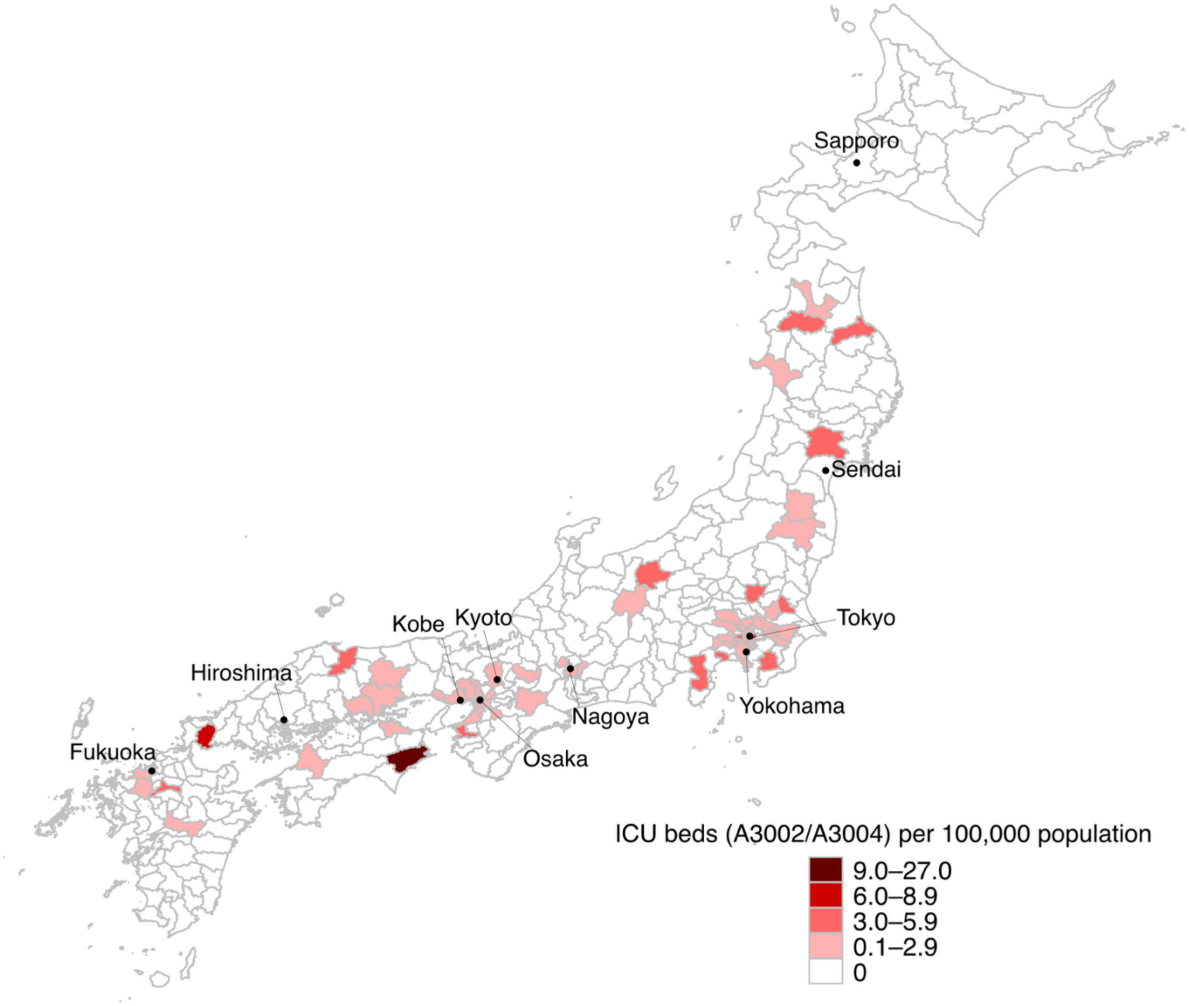

Secondary medical areas without ICU beds (A3002/A3004) are shown in white. The color intensity reflects ICU bed (A3002/A3004) density per 100,000 population.
ICU = intensive care unit

Supplementary Figure 6. Patient Flowchart for the ICU Admission Cohort

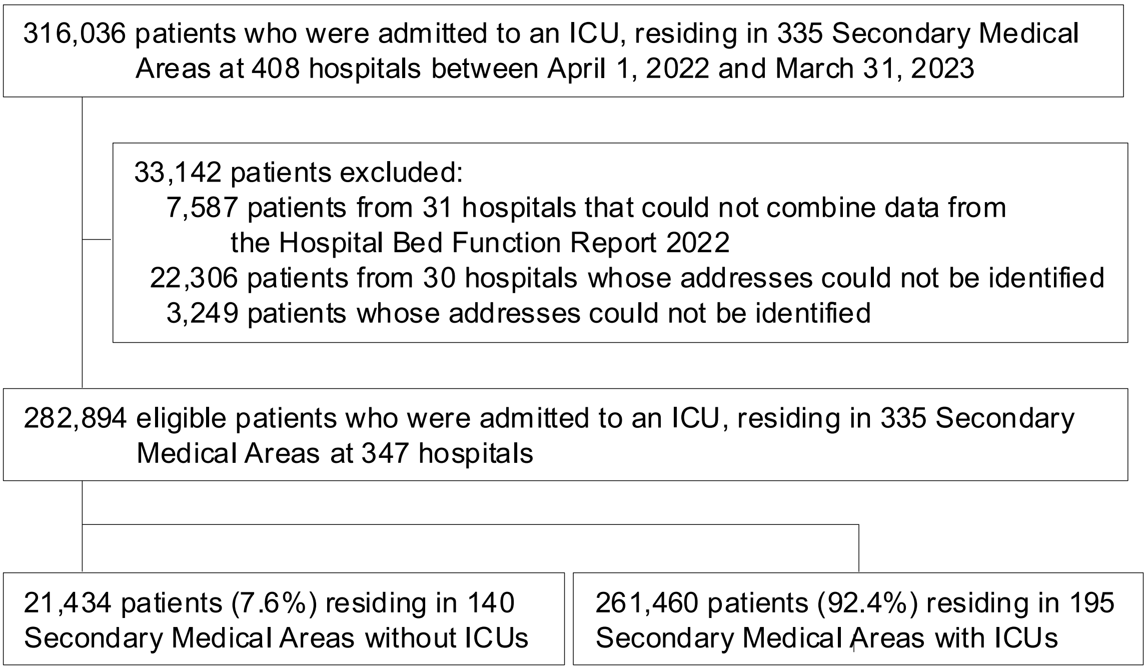

ICU = intensive care unit

Supplementary Figure 7. Geographic Distribution of SMA-level median travel distance in the ICU-admitted cohort

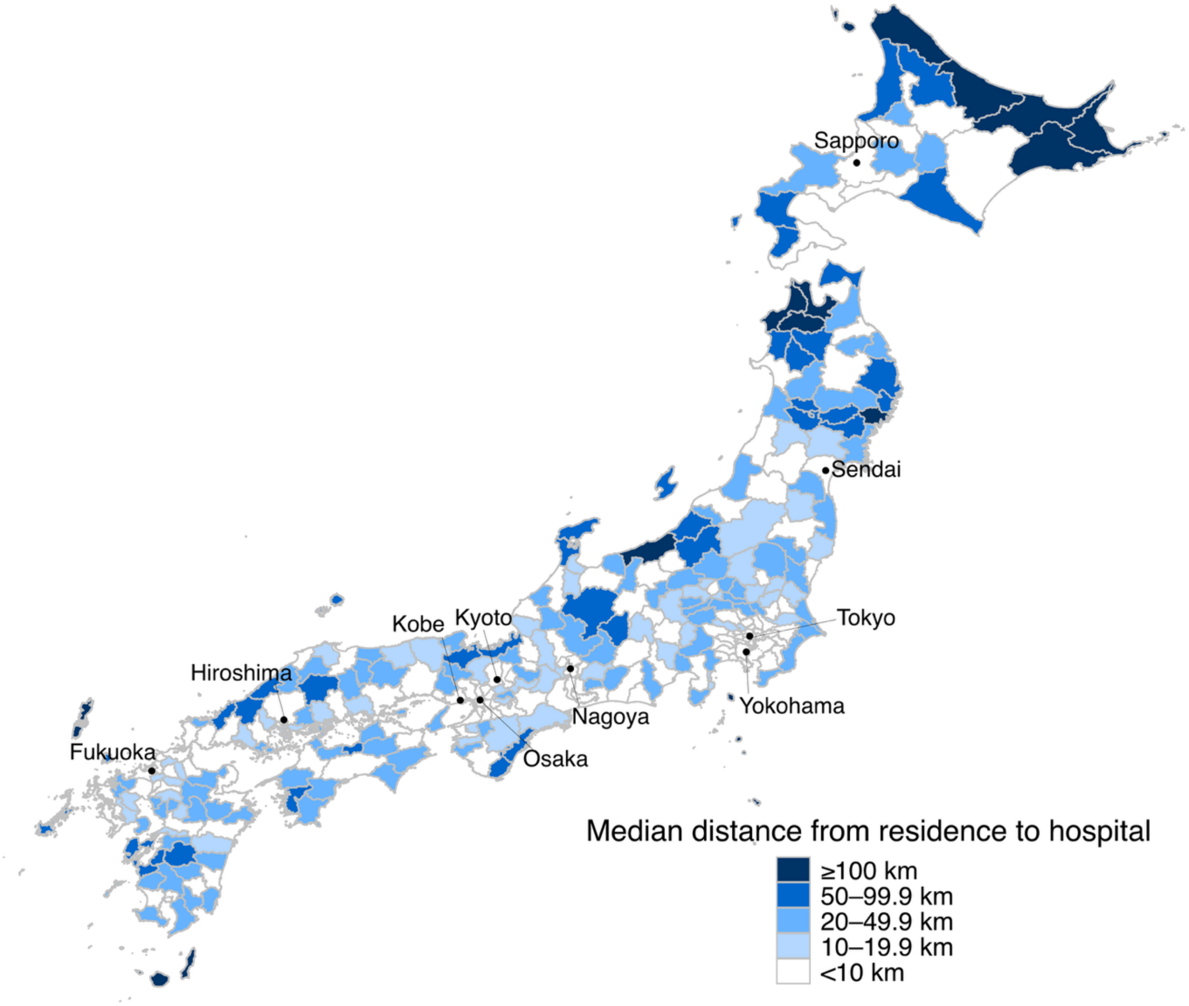

SMA = secondary medical area; ICU = intensive care unit.

Supplementary Figure 8. **Geographic Distribution of SMA-level mean unadjusted in-hospital mortality in the ICU-admitted cohort**

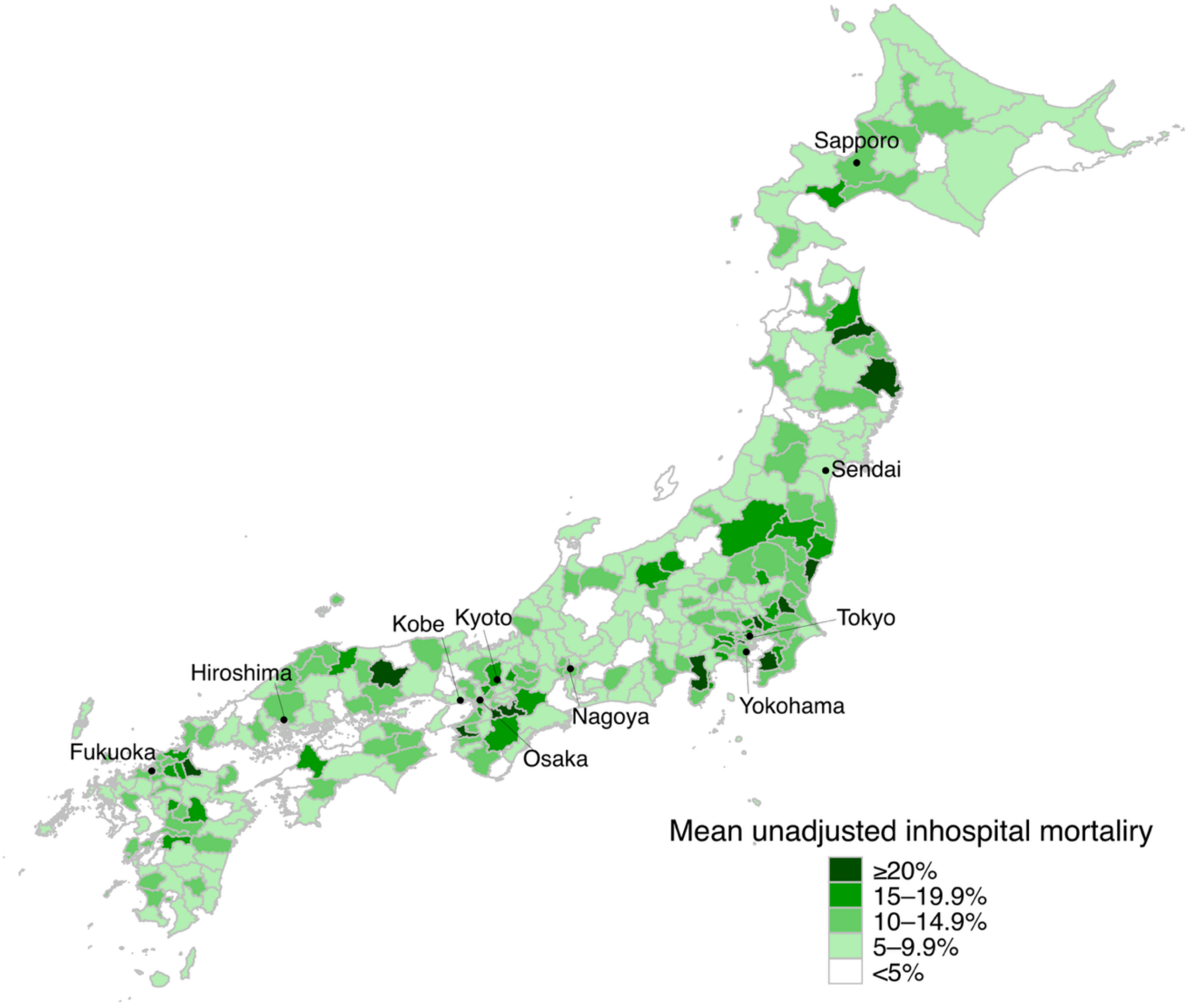

SMA = secondary medical area; ICU = intensive care unit.

Supplementary Figure 9. Patient Flowchart for the Critically Ill Patient Cohort

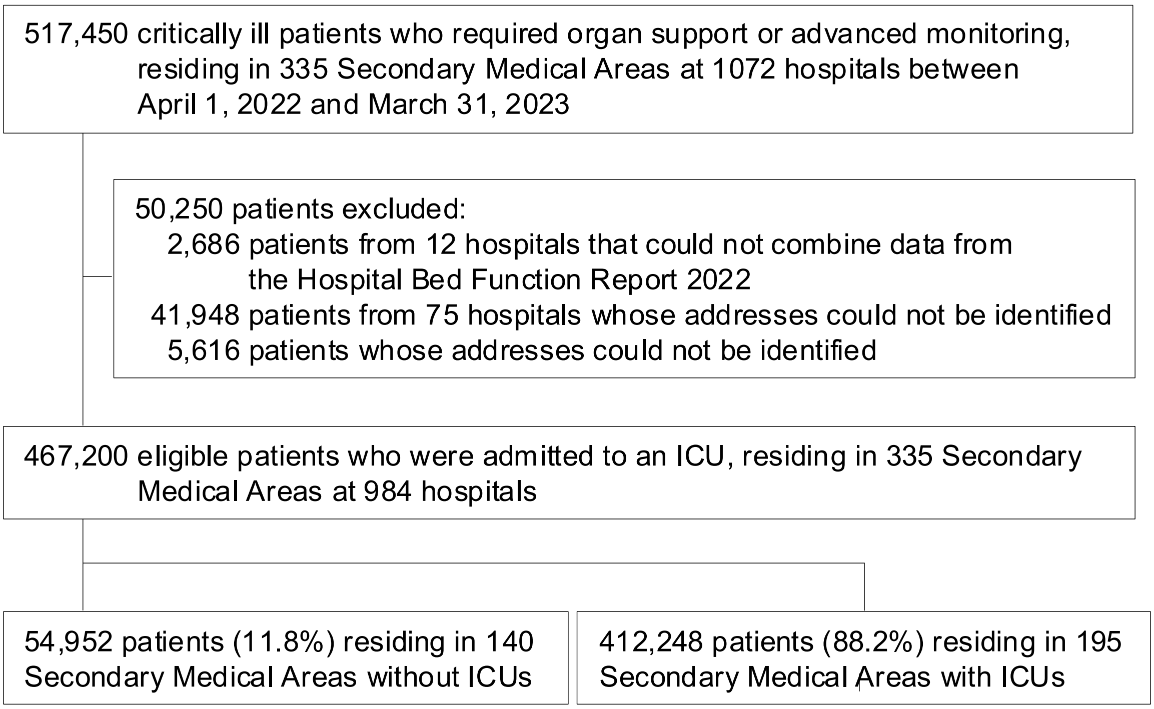

ICU = intensive care unit

Supplementary Figure 10. **Geographic Distribution of SMA-level median travel distance in the critically ill cohorts**

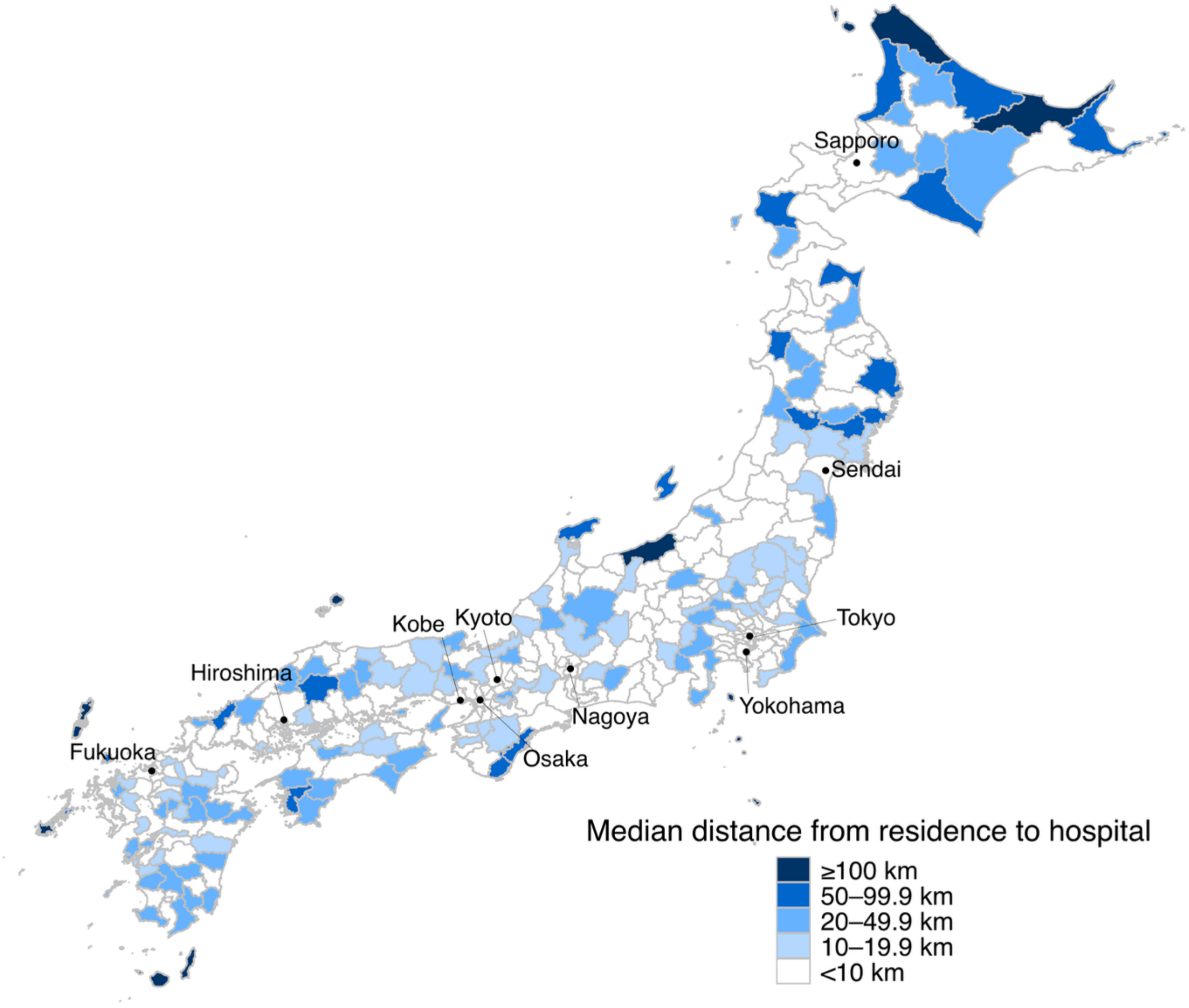

SMA = secondary medical area

Supplementary Figure 11. Geographic Distribution of SMA-level mean unadjusted ICU admission in the critically ill cohorts

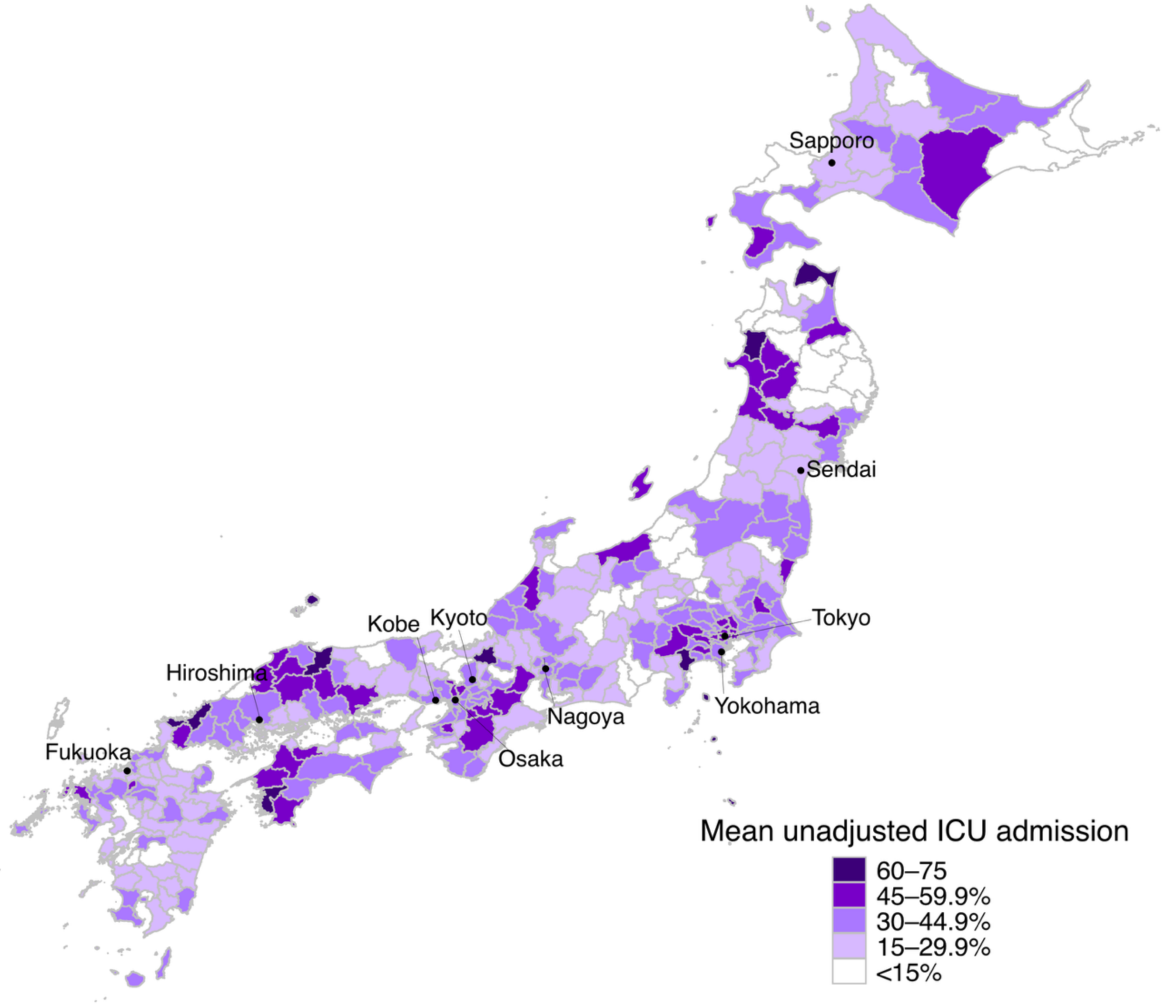

SMA = secondary medical area

Supplementary Figure 12. Geographic Distribution of SMA-level mean unadjusted in-hospital mortality in the critically ill cohorts

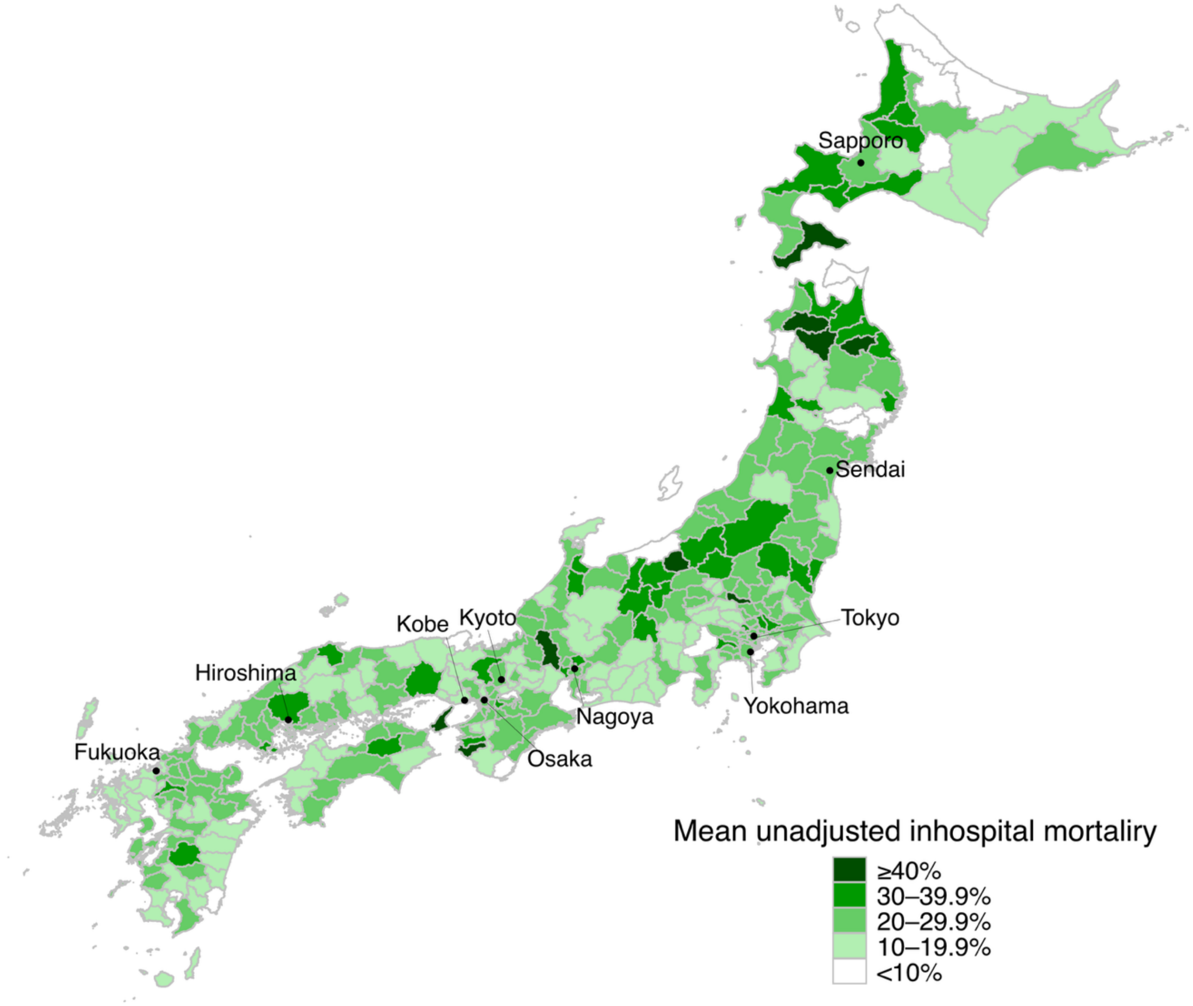

SMA = secondary medical area
